# Mesoporous TiO_2_ and Fe-containing TiO_2_ prepared by solution combustion synthesis as catalysts for the photodegradation of paracetamol

**DOI:** 10.1007/s11356-024-33575-5

**Published:** 2024-05-17

**Authors:** Nadia Grifasi, Fabio Alessandro Deorsola, Debora Fino, Marco Piumetti

**Affiliations:** Department of Applied Science and Technology, Polytechnic of Turin, Corso Duca Degli Abruzzi, 24, 10129 Turin, Italy

**Keywords:** Iron-containing titania, Defective structure, Wastewater treatment, Emerging contaminants, UV photodegradation, Photo Fenton-like process

## Abstract

Water pollution due to emerging contaminants, e.g., pharmaceuticals, is one of the most frequently discussed issues. Among them, paracetamol received great attention due to its physico-chemical properties, persistence, and adverse environmental effects. Different techniques were employed for its degradation and, among them, photodegradation is considered one of the most suitable to pursue the aim. This work aimed to synthesize mesoporous TiO_2_, even with the presence of iron, through a one-pot method, with an enhanced ability to abate paracetamol. Precisely, pure and iron-containing (3.5 wt%) TiO_2_ were successfully obtained employing an uncommon procedure for this kind of material, mainly solution combustion synthesis (SCS). Moreover, a traditional hydrothermal method and a commercial Degussa P25 were also investigated for comparison purposes. The samples were characterized through N_2_-physisorption at − 196 °C, XRD, XPS, EDX, DR UV-Vis, and FESEM analysis. The catalytic activity was investigated for the abatement of 10 ppm of paracetamol, under UV irradiation in acidic conditions (pH = 3) and in the presence of H_2_O_2_. As a whole, the best-performing catalysts were those obtained through the SCS procedure, highlighting a complete removal of the organic pollutant after 1 h in the case of Fe/TiO_2__SCS, thanks to its highly defective structure and the presence of metal Fe. To better investigate the performance of both pure and Fe-containing SCS samples, further oxidation tests were performed at pH = 7 and in the absence of H_2_O_2_. Noteworthy, in these conditions, the two samples exhibited different behaviors, highlighting different mechanisms depending on the presence or absence of iron in the structure. Finally, a kinetic study was conducted, demonstrating that a first order is suitable for its abatement.

**Graphical abstract:**

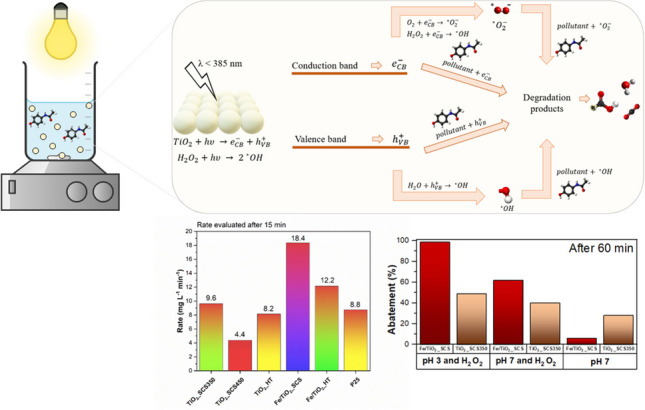

**Supplementary Information:**

The online version contains supplementary material available at 10.1007/s11356-024-33575-5.

## Introduction

Water is one of the most important and valuable resources around the world. It is crucial for life and other beneficial purposes, i.e., agricultural, industrial, and so on. Unfortunately, it is well known that water pollution is one of the most frequently discussed issues in recent years. In this context, industrial effluents are the major source of toxins for groundwater (Kumar 2013). Indeed, production processes release various types of contaminants into water and the environment, causing harmful effects on the ecosystem and human health. In the last decades, researchers have focused attention on “emerging contaminants” which are substances of actual or potential threat to human health or the environment. This class includes pharmaceuticals, personal care products, chemicals used in packaging products, synthetic organic dyes, etc. (Bo et al. 2016). Among pharmaceuticals, paracetamol is widely used since it is an analgesic and antipyretic drug that can temporarily relieve mild-to-moderate pain and fever (Samal et al. 2022). Unfortunately, in the last years, the extent of its consumption provoked an increase in its concentration in wastewater (Adeleye et al. 2022; Waleng and Nomngongo 2022), varying from nanograms per liter in aquatic environments (Al-Kaf et al. 2017) to milligrams per liter in hospital wastewater (Macías-García et al. 2019). Moreover, different classes of pharmaceuticals detected in water systems cause serious adverse effects on the ecosystem (Hernández-Tenorio et al. 2022; Szopińska et al. 2022; Waleng and Nomngongo 2022). Specifically, even if paracetamol is not highly persistent in the environment, it could provoke some dangerous effects on different species living in aquatic environments. Moreover, its decomposition into other compounds, i.e., 4-aminophenol, is reported to have significant nephrotoxicity and teratogenic effects (Wu et al. 2012; Agarwal 2022).

Ecopharmacology is a novel field that investigates the deleterious environmental impacts of pharmaceuticals and their metabolites (Arun et al. 2022; Kayode-Afolayan et al. 2022), which include impacts on biodiversity, persistence, and bioaccumulation across trophic levels in the environment (Kayode-Afolayan et al. 2022). For these reasons, it is crucial and urgent to employ strategies for water remediation. Among them, conventional and emerging technologies have been still used, i.e., adsorption, biological treatment, and catalytic process (Phoon et al. 2020). Adsorption is low-cost, simple, and highly effective for the removal of contaminants from waste streams. Many studies report the use of natural zeolites, biochar, or activated carbon (AC) for removing synthetic organic dyes, pharmaceuticals, etc. (Phoon et al. 2020; Ndoun et al. 2021; Dosa et al. 2022). Specifically, AC is the most used for the adsorption of paracetamol (Macías-García et al. 2019; Mansouri et al. 2021). Unfortunately, this technique only can transfer the pollutants from the liquid phase to the solid one, and sometimes, the cost of the adsorbent could be high, i.e., in the case of activated carbon (Mohan and Pittman 2006). For instance, in a report published by Global Trade in March 2023, the price of activated carbon amounted to 6401 $ ton^−1^ due to the very expensive activation processes (IndexBox 2023a). Another technology exploits the use of some microorganisms that are capable of using paracetamol as a carbon and energy source and are also capable of degrading and converting it to non-toxic compounds (Al-Kaf et al. 2017; Rios-Miguel et al. 2022). However, its removal could be quite low since it may resist biological degradation (Macías-García et al. 2019). Thus, considering the physico-chemical properties of paracetamol, conventional treatments demonstrate inefficiency for its removal (Pacheco-Álvarez et al. 2022).

Recently, advanced oxidation processes (AOPs) have been considered effective for this purpose, since it is possible to degrade a wide range of pollutants by generating in situ highly reactive radicals, i.e., hydroxyl radicals. The major advantage of AOPs is their capability to destroy organic pollutants without transferring them to another phase and reduce/avoid the presence of by-products (Wadhah 2017). Among them, the technology that employs TiO_2_ as a photocatalyst, with the combined effect of UV radiation and H_2_O_2_, is extremely efficient for complete degradation, above all in acidic media. It exhibits some advantages since hydrogen peroxide is inexpensive, not dangerous, easy to handle, and decomposes easily into water and oxygen. Likewise, TiO_2_ received extensive attention since it is chemically stable, non-toxic, and cost-effective with a price amounting to 3000 $ ton^−1^ (Yang et al. 2008; Borges et al. 2015; Wadhah 2017; IndexBox 2023b). To enhance the catalytic properties of this oxide toward paracetamol, many studies report the possibility of introducing different heteroatoms, mainly iron (Cifci et al. 2017; Yap et al. 2020; de Moura et al. 2021). This element is earth-abundant and non-toxic (Gervasi et al. 2023) and its effect is crucial since it can be determinant for an efficient degradation (Trovó et al. 2012). Indeed, it is well known that the presence of iron, in particular Fe^2+^, and the synergy with H_2_O_2_ can enhance the mineralization of the paracetamol, mostly through the (Photo)Fenton process, which is another method belonging to AOPs.

The methods for the synthesis of titania, with the possible presence of iron, are fairly well known and the conventional ones mainly involve hydrothermal treatments or pyrolysis (in the case of commercial P25), to confer well-defined properties. However, these procedures are extremely time-consuming and, above all, impact the environment as they require the use of various chemicals, including templating agents, acids, and so on. For this reason, in this paper, the attention is focused on the fabrication of advanced materials via green and sustainable paths to accomplish chemical circularity. The design and preparation methods play a key role in determining the structural, surface characteristics, and catalytic properties of the photocatalysts. In this regard, the solution combustion synthesis (SCS) technique is a relatively novel, cost-effective, and efficient method for the bulk production of nano and meso-structured materials (Siddique et al. 2022). SCS-fabricated metal oxides are of great technological importance in photocatalytic, environmental, and energy applications. To date, the SCS route has been employed to produce a large variety of solid materials. However, in the literature, there is a lack of use of this technique for the preparation of titania oxide. Thus, this work explored the possibility of obtaining highly active mesoporous TiO_2_ and Fe-containing TiO_2_ synthesized through a modified SCS procedure, which is uncommon for this kind of material. We intended to promote the employment of a synthesis procedure that is scarcely used for this application. The strengths and advantages to employing SCS are manifold; i.e., it is more sustainable, green, cost-effective, and non-time-consuming compared to the conventional ones, with less impact on the environment. Therefore, the aim of this work was related to propose a novel and optimized strategy to obtain remarkable properties that are crucial in the photocatalytic application field, and, in particular, for the degradation of paracetamol.

## Materials and methods

### Chemicals

Different titanium oxides were synthesized through hydrothermal procedure (labeled as HT from now on) and solution combustion synthesis (named SCS from now on). All the reagents employed for the preparation of catalysts were used directly without any further purification: titanium isopropoxide (97%, Sigma-Aldrich); Pluronic 123 (P123, poly(ethylene glycol)-poly-(propylene glycol)-poly(ethylene glycol); average Mn ∼ 5800; Sigma-Aldrich); acetic acid solution (ACS reagent, > 99.8%, Sigma-Aldrich); Milli-Q water; urea (ACS reagent, 99–100.5%, Sigma-Aldrich), ethanol (Reag. Ph. Eur, VWR Chemicals); FeCl_3_·6H_2_O (Reagent grade, 97%, Sigma Aldrich); paracetamol (European Pharmacopoeia Reference Standard, qualification CRS).

### Catalyst preparation through hydrothermal procedure

For the preparation of mesoporous titania, a micelle template-assisted sol–gel procedure was employed by introducing some changes in the methods reported in the literature (Piumetti et al. 2014; Freyria et al. 2017; Blangetti et al. 2023). Briefly, two different solutions containing titanium(IV) isopropoxide and acetic acid solution (solution A) and P123 and ethanol (solution B) were prepared separately and then mixed. Subsequently, the resulting mixture was stirred for 24 h at room temperature and then transferred into a stainless-steel autoclave (Berghof) thoroughly sealed, where the hydrothermal treatment occurred at 85 °C for 48 h. After the natural temperature cooled down, the solid product was centrifuged, dried at 80 °C for 24 h, and calcinated in air at 450 °C for 4 h with a ramp of 1.8 °C min^−1^. The sample obtained was denoted as TiO_2__HT. The same procedure was adopted for the preparation of Fe-containing TiO_2_ (3.5 wt% nominal Fe content), by adding a proper amount of FeCl_3_·6H_2_O to solution A. The sample containing Fe was denoted Fe/TiO_2__HT.

### Catalyst preparation through solution combustion synthesis

For the preparation of titania through SCS, an ex novo procedure was employed. A proper amount of titanium(IV) isopropoxide (used as a precursor) was dissolved in water under stirring at room temperature until a complete solution was obtained. Urea was employed as fuel and added to the solution to carry on the exothermic redox reaction. Precisely, the fuel-to-oxider ratio was fixed at a value of 0.7. Finally, the solution was transferred to a crucible and placed in an oven, where the reaction occurred at 450 °C for 4 h with a ramp of 5 °C min^−1^. To investigate the influence of calcination temperature on the catalyst’s performance, another sample was synthesized by carrying out the reaction at 350 °C for the same time as in the previous case. The obtained samples were denoted TiO_2__SCS450 and TiO_2__SCS350, respectively. The best performing was also modified with 3.5 wt% of nominal Fe content, by employing a one-pot SCS procedure, adding the proper amount of FeCl_3_·6H_2_O to the initial solution, and following the same procedure described above. The obtained sample was denoted Fe/TiO_2__SCS.

### Catalyst characterization

Textural properties, e.g., the specific surface area (SSA), the total pore volume (VTP), and the pore diameter (Dp), were evaluated by performing N_2_-physisorption at − 196 °C in a Micromeritics Tristar II 3020 (v1.03, Micromeritics Instrument Corp., Norcross, GA, USA). Prior to start, the powder samples were outgassed under N_2_ flow at 200 °C for 2 h to remove impurities on their surface (e.g., moisture). Brunauer–Emmett–Teller (BET) method and Barrett–Joyner–Halenda (BJH) (during desorption phase) method were employed to estimate the SSA (m^2^ g^−1^) and the VTP (cm^3^ g^−1^) and Dp (nm), respectively.

The crystalline structure of the samples was investigated using powder X-ray diffraction analysis (XRD). The patterns were collected with an X’Pert Philips PW3040 (Malvern Panalytical Ltd., Malvern, UK) diffractometer using Cu Kα radiation (2θ range = 20°–90°; step = 0.05° 2θ; time per step = 0.2 s). The collected diffraction peaks were analyzed according to the Powder Data File database (PDF-2004, International Centre of Diffraction Data), and the average crystallite size was evaluated using Scherrer’s formula *D* = 0.9λ/(*b* cos θ), where λ is the wavelength of the Cu Kα radiation, *b* is the full width at half maximum (in radians), 0.9 is the shape factor considered for spherical particles, and θ is the angle of the diffraction peaks (Cullity 1994).

The elemental composition and oxidation states of elements at the surface of the materials were evaluated through X-ray photoelectron spectroscopy (XPS) in a PHI 5000 Versa probe apparatus (Physical Electronics Inc. PHI, Chanhassen, MN, USA) using the following conditions: band-pass energy of 187.85 eV, a 45° take-off angle, and a 100.0-μm diameter X-ray spot size.

The morphology of the samples was studied through field emission scanning electron microscopy analysis (FESEM, Zeiss MERLIN, Gemini-II column, Oberkochen, Germany) using an extra high tension (EHT) of 3 kV, a working distance (WD) of about 3 mm, and a probe intensity of 115 pA.

EDX analysis was performed on the same apparatus to evaluate the percentage of iron present in the samples. The analysis was carried out in three different points, to verify if the material was homogenous.

Optical properties of TiO_2_ and Fe-containing TiO_2_ were studied through the diffuse reflectance (DR) UV–Vis spectroscopy, employing a double-beam spectrophotometer (Varian Cary 5000, Varian, Inc., Palo Alto, CA, USA) integrated with a sphere. The spectra were acquired in the range of 200–600 nm by introducing 500 mg of powder into the sample holder. Diffuse reflectance UV–Vis spectra were recorded in reflectance mode (*%R*) and then the Kubelka–Munk function (*F*(*R*∞)) was used to calculate the band gap energy through Tauc’s Plot.

#### Photocatalytic tests and kinetic study

The abatement tests were carried out in acidic conditions (pH≈3) and the presence of hydrogen peroxide, under long-wave UV irradiation equal to 365 nm, belonging in the range of UV-A (315–400 nm) (Photo-Fenton reaction). Before starting, preliminary considerations were made to optimize the quantity of H_2_O_2_ in order to avoid any interference of its UV–VIS spectra with the band of paracetamol. Subsequently, the suitable amount of hydrogen peroxide was added to the solution containing H_2_SO_4_ (used to reach the pH of 3) and 10 mg L^−1^ of paracetamol under stirring to obtain a complete dissolution of the pollutant. The first spectrum at time *t* = 0 was acquired through a spectrophotometer (Hach Lange DR5000) in the wavelength range of 200–400 nm. After that, 2 g L^−1^ of titania was added under stirring and UV irradiation (Spectroline, long wave ultraviolet 365 nm). At the prescribed time, an aliquot of the solution was taken with a syringe and filtered to remove the catalyst. After that, the spectra were acquired in the same wavelength range of 200–400 nm and the procedure continued until the abatement of the pollutant was completed. Further tests were carried out under the exclusion of light in dark conditions (Fenton reaction) by following the same procedure described above. Finally, the best-performing catalysts were also tested in “greener” conditions compared to the previous ones. In particular, photocatalytic tests were performed at pH close to the neutrality and with and without the presence of H_2_O_2_, to better investigate the performance of the samples under varying operating conditions.

Moreover, to better understand the behavior of the catalysts and the mechanism involved in the removal of paracetamol, kinetic studies were carried out by implementing pseudo-first-order (PFO) and pseudo-second-order (PSO) kinetic models. The linearized equations of PFO and PSO are described in Eqs. 1 and 2 respectively, where *C*_*t*_ (mg L^−1^) is the concentration of pollutant at a generic time *t* (min), *C*_0_ is the initial concentration of pollutant (mg L^−1^), and *k*_1_ (mg L^−1^) and *k*_2_ (L mg^−1^ min^−1^) are the rate constants of PFO and PSO models.

PFO:

1 $${\text{ln}}\left({C}_{t}\right)={\text{ln}}\left({C}_{0}\right)- {k}_{1}\cdot t$$

PSO:

2 $$\frac{1}{{C}_{t}}=\frac{1}{{C}_{0}}+ {k}_{2}\cdot t$$

An important parameter used to discriminate the most suitable kinetic model was the half-life *t*_1/2_, corresponding to the time required to halve the pollutant concentration and calculated by substituting *C*_*t*_ with 0.5 *C*_0_ in Eqs. 1 and 2, respectively.

## Results and discussion

### Physico-chemical characterization

**Fig. 1:**
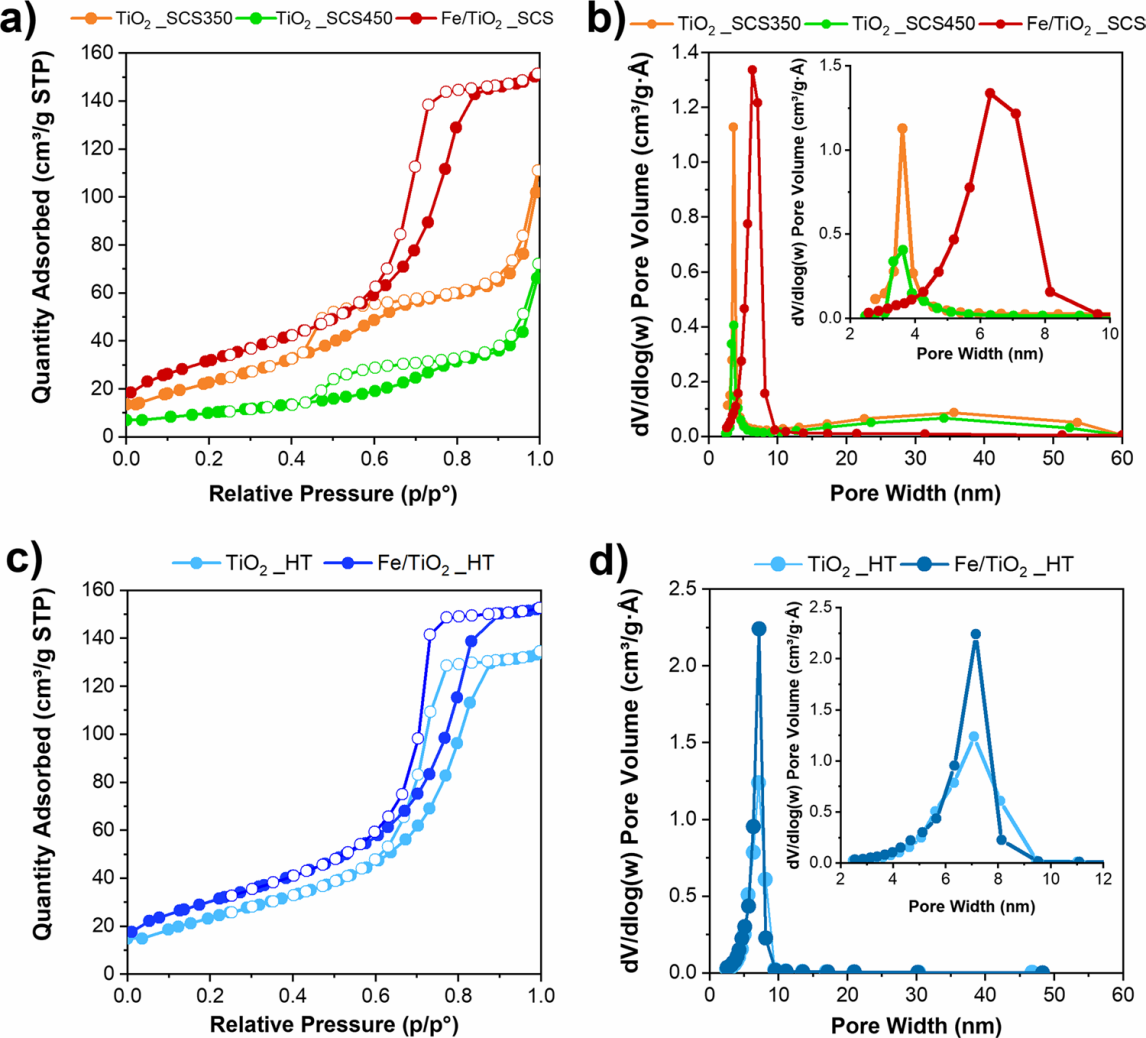
Adsorption–desorption isotherms of samples synthesized through SCS (**a**) and HT (**c**) methods and their respective pore size distributions (**b**, **d**)

Figure 1 reports the adsorption–desorption isotherms and the pore size distributions of the synthesized samples and Fig. S1 shows the isotherms of P25, whereas Table 1 shows the values of the SSA (m^2^ g^−1^), the VTP (cm^3^ g^−1^), and the average size of pore diameter (Dp, nm) obtained from N_2_ physisorption at − 196 °C of all the synthesized materials.

**Table 1:** Textural properties of the prepared and commercial titania oxides, calculated from N_2_ physisorption at − 196 °C

**Sample**	**SSA**^a^ [**m**^**2**^ **g**^**−1**^]	**Vp**^b^ [**cm**^**3**^ **g**^**−1**^]	**Dp**^c^ [**nm**]
TiO_2__SCS350	88	0.18	6.6
TiO_2__SCS450	38	0.11	8
Fe/TiO_2__SCS	118	0.24	6
TiO_2__HT	84	0.21	6.4
Fe/TiO_2__HT	113	0.24	6

^a^Specific surface area (SSA) evaluated according to the Brunauer–Emmett–Teller (BET) method

^b^Total Pore Volume (VTP) evaluated according to the Barrett–Joyner–Halenda (BJH) method during the desorption phase

^c^Average pore diameter (Dp) evaluated according to the Barrett–Joyner–Halenda (BJH) method during the desorption phase

As a whole, the textural properties of synthesized materials depended on the preparation methods. TiO_2_ samples obtained through SCS (**a**) exhibited two hysteresis loops at high and low relative pressure. This phenomenon could be related to the presence of pores belonging to different range sizes. Precisely, isotherms exhibited adsorption hystereses associated with type IV and, according to IUPAC, this loop can be ascribed to type H4, referred to a broad pore size distribution with smaller pores accessible (i.e., presence of microporosity). In fact, by analyzing the pore size distributions (**b**), it appeared clear the presence of bimodal distribution, with pores belonging to ca. 4 nm and ca. 35 nm. Moreover, the value of SSA was found to be extremely dependent on the calcination temperature. Indeed, the TiO_2__SCS350 exhibited a higher surface area value (88 m^2^ g^−1^), compared to TiO_2__SCS450 (38 m^2^ g^−1^), demonstrating that temperature had a crucial impact on textural properties. On the other hand, the sample containing iron, named Fe/TiO_2__SCS, exhibited a completely different behavior. Precisely, as can be seen in Fig. 1**a**, isotherms exhibited adsorption hystereses associated with type IV. According to IUPAC, this loop can be ascribed to type H1, often associated with porous materials consisting of well-defined cylindrical-like pore channels. By analyzing the pore size distributions (**b**), Fe/TiO_2__SCS showed a well-defined and narrow pore size distribution of about 4.0–8.0 nm, whereas, regarding the samples obtained through the hydrothermal (HT) method, both Fe/TiO_2__HT and TiO_2__HT exhibited the same isotherms profile (**c**) and pore size distribution (**d**) of Fe/TiO_2__SCS. Finally, it is noteworthy to observe that both TiO_2__HT and TiO_2__SCS350 showed comparable SSA values, equal to 84 m^2^ g^−1^ and 88 m^2^ g^−1^, respectively, indicating the possibility of reaching high values even with a simple and cost-effective method, which higher compared to that of P25 (see Table S1). Concerning iron-containing materials, Fe/TiO_2__SCS and Fe/TiO_2__HT showed the highest SSA values among all the samples, equal to 118 m^2^ g^−1^ and 113 m^2^ g^−1^, respectively, highlighting that the presence of iron could enhance the specific surface area.

**Fig. 2:**
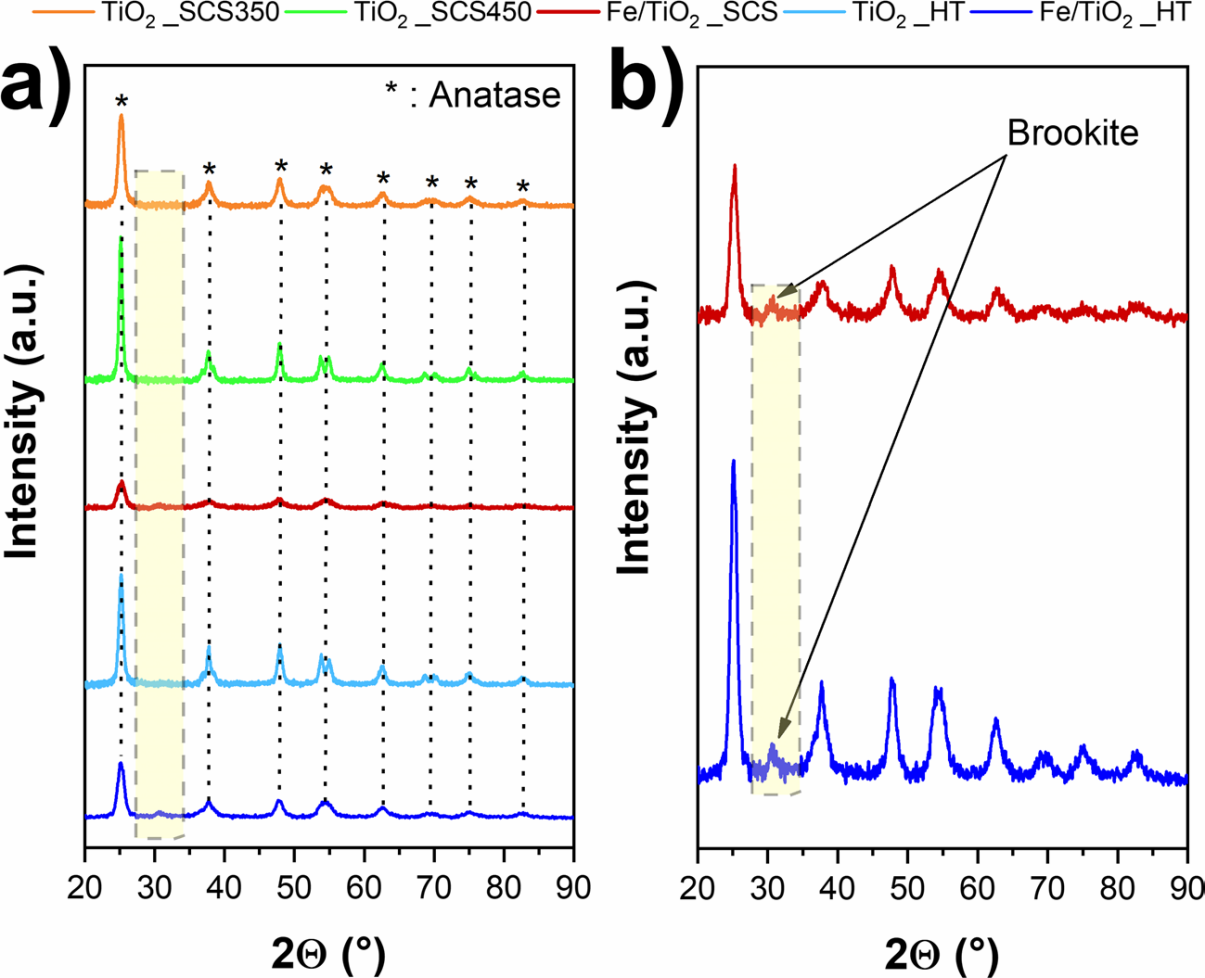
X-ray diffraction patterns of synthesized samples (**a**) and a magnification of Fe/TiO_2__SCS and Fe/TiO_2__HT diffractograms (**b**)

Figure 2 reports the diffraction patterns of all the synthesized samples, whereas Table 2 reports the type of crystalline phases detected and the average crystallite size for all samples.

**Table 2:** Crystalline phases detected and the average crystallite size for all synthesized samples

**Sample**	**Crystalline Phase Detected**			**Crystallite size**
	**Anatase**	**Brookite**	**Rutile**	**[nm]**
TiO_2__SCS350	°			11
TiO_2__SCS450	°			16
Fe/TiO_2__SCS	°	°		5
TiO_2__HT	°			13
Fe/TiO_2__HT	°	°		7

As a whole, all the synthesized samples exhibited the presence of anatase, with the most intense peak at 2θ = 25.31° corresponding to (1 0 1)-type planes, in accordance with the reference patterns in the database (ref. 01–071-1166). The presence of rutile was detected only in the P25 sample (see Fig. S2), following the literature (Freyria et al. 2017; Blangetti et al. 2023). It was noteworthy to observe that only the samples containing iron, named Fe/TiO_2__SCS and Fe/TiO_2__HT, exhibited a peak at 2θ = 30.81°, which is ascribed to the presence of Brookite (ref. 01–071-1166) and corresponding to (2 1 1)-type plane. Its presence could be justified both by considering the calcination temperature and the pH of the solution during the synthesis procedure. Indeed, the formation of brookite is favored by synthesis temperatures below 600 °C and acid pH (Tsega and Dejene 2017; Allen et al. 2018). Both conditions were in agreement with the synthesis procedures adopted in these cases, as the calcination temperature was below 600 °C, while the acid pH could be due to the presence of the iron chloride used as a precursor. Thus, the combination of these parameters may have determined the optimal conditions for the formation of brookite as a polymorph. Concerning the samples obtained through the SCS method, mainly TiO_2__SCS350 and TiO_2__SCS450, the effect of calcination temperature appeared evident. Precisely, by performing a thermal treatment at 450 °C, it was evident a greater crystallinity of the sample, given by well-defined and narrow peaks. Indeed, the characteristic peak of anatase (2θ = 25.31°) had a higher intensity for TiO_2__SCS450 than that obtained for TiO_2__SCS350. Moreover, in TiO_2__SCS450, it was possible to clearly distinguish the two pairs of peaks at 53.88°–55.07° and 68.76°–70.30°, while in sample TiO_2__SCS350 they appeared to collapse into a single broader peak at 54.39° and 69.72°, respectively. On the other hand, comparing the samples obtained at the same temperature, i.e., TiO_2__SCS450 and TiO_2__HT, they showed the same peak characteristics, highlighting the effect of temperature even with different synthesis procedures. Finally, comparing the diffractograms for the iron-containing samples, it appeared that Fe/TiO_2__SCS showed less intense and broad peaks compared to Fe/TiO_2__HT, attributable to a greater disorder of the structure, as would be expected from the synthesis procedure. Moreover, the difference in intensities and broader or narrower peaks also indicated different crystallite sizes, as reported in Table 2. Except for the commercial P25 sample (Table S1), all the synthesized ones showed smaller crystallite average dimensions. Precisely, the bare materials exhibited similar values equal to 11 nm, 13 nm, and 16 nm for TiO_2__SCS350, TiO_2__HT, and TiO_2__SCS450, respectively, whereas the samples containing iron showed the smallest dimensions, specifically 5 and 7 nm for Fe/TiO_2__SCS and Fe/TiO_2__HT, thus assuming probable greater reactivity due to the smaller crystallite size.

**Fig. 3:**
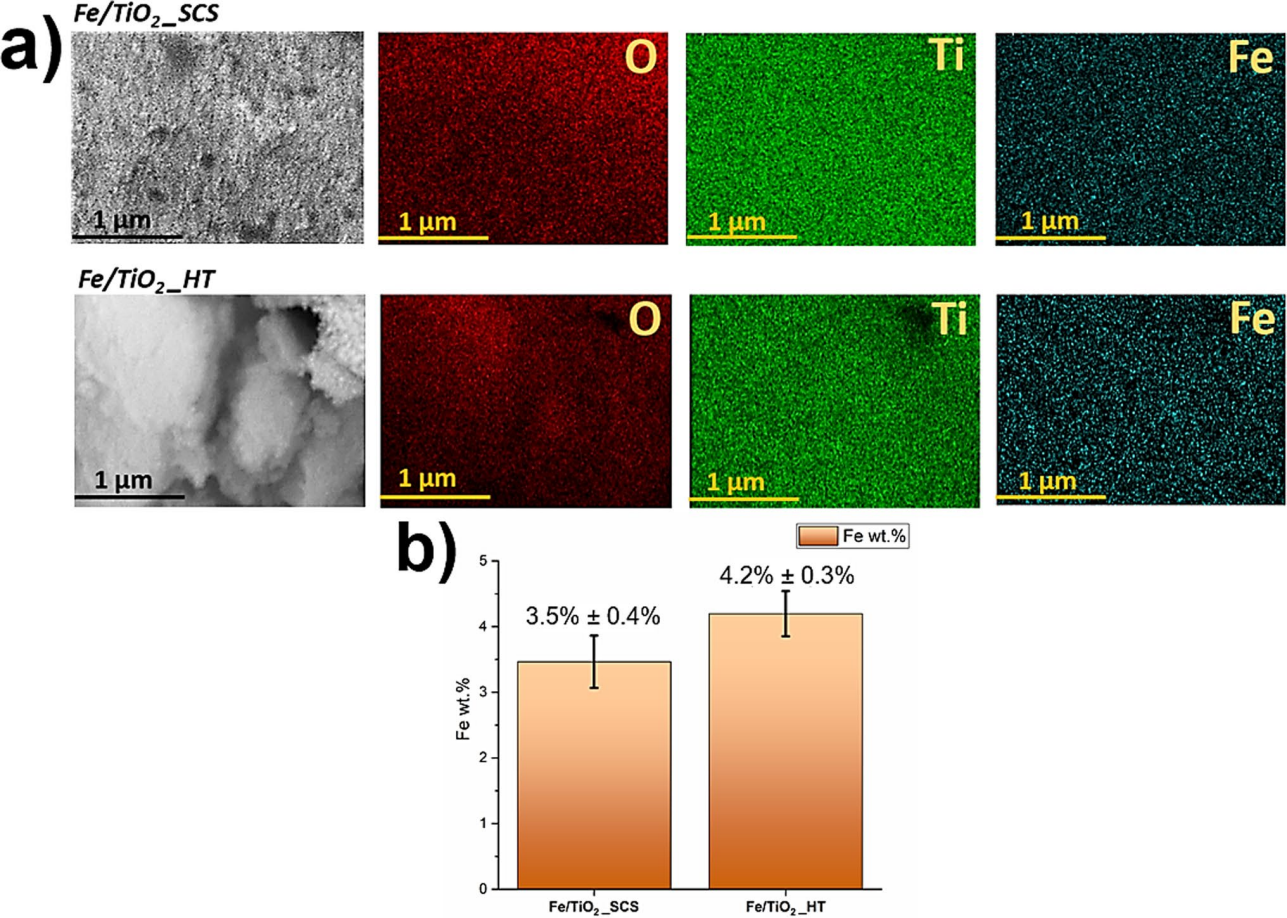
EDX maps (**a**) and iron content (**b**) detected in the Fe/TiO_2__SCS and Fe/TiO_2__HT samples

EDX analysis was performed on Fe-containing samples to obtain the effective amount of iron present in the structure. The results were reported in Fig. 3, where it was possible to observe a good dispersion of Fe in both samples from the EDX maps (**a**), whereas the amount was slightly different (**b**). Specifically, Fe/TiO_2__SCS contained 3.5 wt% with a standard deviation of 0.4%, whereas the sample obtained through the hydrothermal method exhibited a higher percentage of iron compared to the first sample, equal to 4.2 wt% with a standard deviation of 0.3%.

**Fig. 4:**
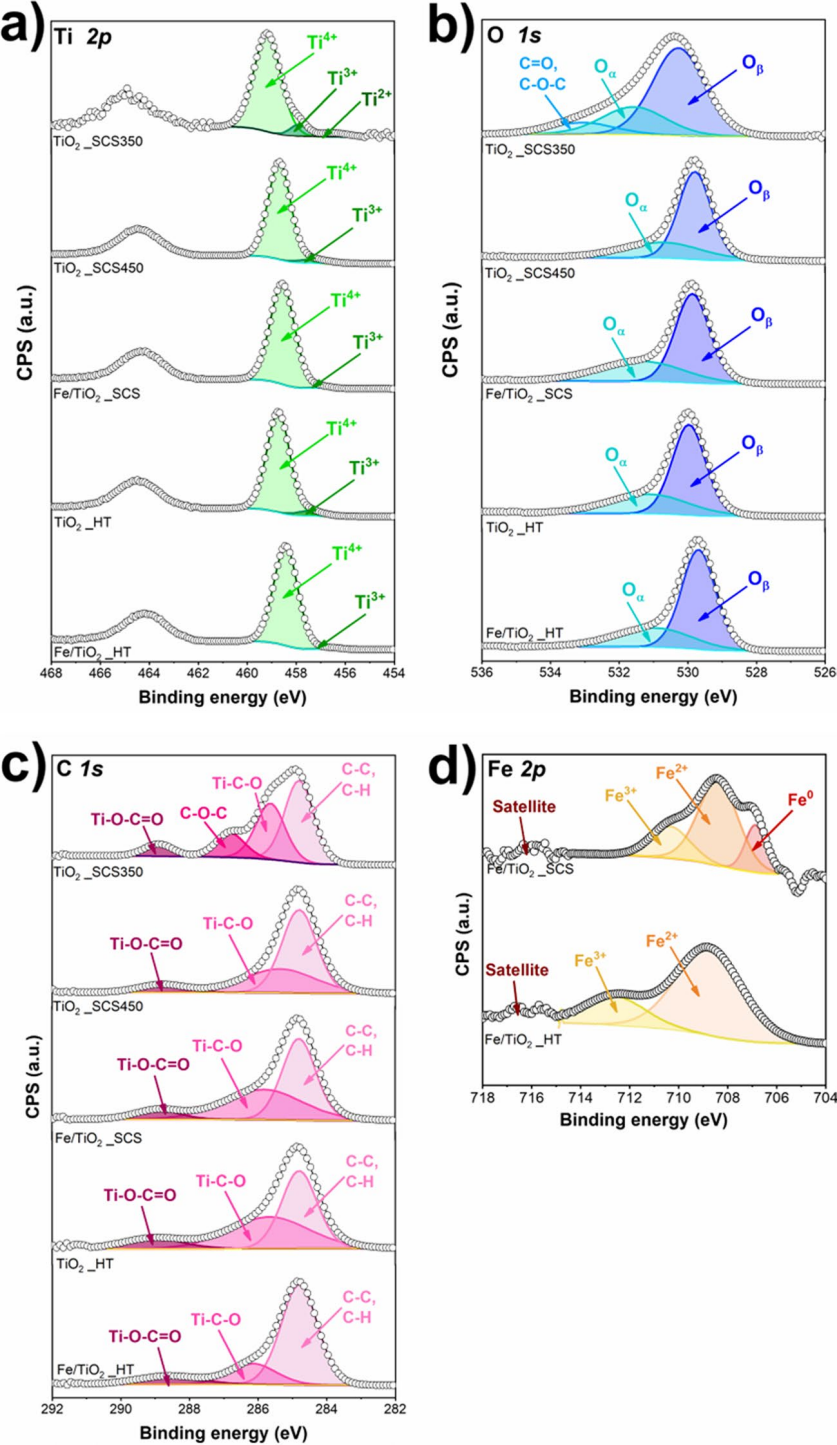
XPS spectra of Ti 2p (**a**), O 1 s (**b**), C 1 s (**c**), and Fe 2p (**d**) of the samples

To better investigate the elemental composition and the oxidation state of the surface species (mainly, Ti, O, Fe, and C elements) of the catalysts, X-ray photoelectron spectroscopy (XPS) measurements were performed. The deconvoluted spectra of Ti, O 1 s, C 1 s, and Fe 2p are reported in Fig. 4, whereas the results related to the relative amount of the species are summarized in Table 3.

**Table 3:** Atomic concentration of the species detected from the spectra deconvolutions

Element	Species detected	TiO_2__SCS350	TiO_2__SCS450	TiO_2__HT	Fe/TiO_2__SCS	Fe/TiO_2__HT
O *1 s*	Lattice	64.2%	68.9%	65.8%	63.9%	69.6%
	OH	25.0%	31.1%	34.2%	36.1%	30.4%
	C = O	10.8%	–	–	–	–
Ti *2p*	Ti^4+^	89.1%	94.7%	94.2%	98.5%	98.6%
	Ti^3+^	6.9%	5.3%	5.8%	1.5%	1.4%
	Ti^2+^	4.0%	–	–	–	–
C *1 s*	Ti–C–O	32.2%	34.5%	42.5%	42.7%	18.6%
	C–O–C	13.8%	–	–	–	–
	Ti–O–C = O, C = O	7.0%	7.4%	10.9%	7.5%	10.2%
	C–C, C–H	47.0%	58.1%	46.6%	49.8%	71.2%
Fe *2p*	Fe^0^	–	–	–	17.3%	–
	Fe^2+^	–	–	–	60.5%	76.4%
	Fe^3+^	–	–	–	22.2%	23.6%

As highlighted from Ti *2p* spectra (**a**), for all the samples, the Ti2p_3/2_ peak was deconvoluted in two components. The first one, located at binding energy between 459.2 and 458.4 eV, was ascribed to Ti^4+^ species. The second peak, located at slightly lower binding energies, precisely between 456.6 and 457.9 eV, was referred to as the Ti^3+^ species. These considerations were also valid for the P25 sample (see Fig. S3) and were consistent with the literature (Peng et al. 2018; Wtulich et al. 2022). It was interesting to observe that the Fe-containing samples exhibited a slight shift toward lower binding energy compared to the pristine samples, probably due to the presence of iron, which modified the chemical surroundings. Surprisingly, by analyzing the Ti2p_3/2_ peak for TiO_2__SCS350, it was possible to observe the presence of another component at lower binding energy, precisely at 456.5 eV which is ascribed to Ti^2+^ species (Peng et al. 2018). It was also important to consider the relative amount of these species. The presence of a high content of Ti^3+^ was detected in TiO_2__SCS350, TiO_2__HT, and TiO_2__SCS450, as reported in Table 3, and could be an index of the defectivity of the structure (Bharti et al. 2016), whereas, in Fe/TiO_2__SCS and Fe/TiO_2__HT, there was a decrease of this species, probably due to the presence of iron which influenced the structure in both cases. Figure 4**b** reports the O *1 s* spectra. From the deconvolution, it was possible to observe the presence of two components for all the samples investigated. The peak located at 529.7–530.3 eV was related to lattice oxygen O_β_ (O^2−^ species) coordinated to Ti^4+^ in the bulk of the structure (Ti–O–Ti) and it was the most intense. The second one was located at higher values of binding energy, between 530.7 and 531.6 eV, and it was related to oxygen vacancies and superficial chemisorbed oxygens, labeled as O_α_ (i.e., O_2_^−^, O^−^, OH^−^) as previously observed in other works (Piumetti et al. 2015; Bhange et al. 2016; Peng et al. 2018; Esposito et al. 2021; Nawaz et al. 2021; Wtulich et al. 2022). For TiO_2__SCS350, even for the oxygen spectra, it can be observed a further component at higher binding energy, mainly at 533.1 eV, ascribed to the presence of C = O or C–O–C species (Wtulich et al. 2022). Considering the relative amount of all species, the most abundant component was lattice oxygen (see Table 3 and Table S2). Moreover, it was noteworthy to highlight that, among the samples, TiO_2__SCS350 and Fe/TiO_2__SCS exhibited the highest amount of defective oxygen species, precisely 35.8% and 36.1% respectively, which may contribute to enhancing the catalytic properties. Precisely, in the Fe/TiO_2__SCS sample, the amount of these oxygens increased probably due to the substitution of Ti^4+^ with Fe^3+^ species, thus generating more oxygen vacancies (Bharti et al. 2016), but at the same time, some Ti^3+^ ions are probably replaced with Fe^3+^, thus decreasing their percentage.

The assignment of the deconvoluted peaks in the carbon spectrum was more complicated due to the large variety of carbon species that may be present. Figure 4**c** reports the C 1 s spectra, by considering the reference peak at 284.8 eV. All the samples investigated exhibited a peak at binding energy ranging from 285.4 to 286.1 eV, which was ascribed to the presence of Ti–C–O (Xing et al. 2014; Roy et al. 2019; Gloria et al. 2023). It was interesting to observe that both the Fe-containing samples exhibited a shift of this peak at higher binding energy, specifically at 285.8 eV and 286.1 eV in the case of Fe/TiO_2__SCS and Fe/TiO_2__HT, respectively. This could be due to the presence of iron in the structure which modified the chemical environment, as previously mentioned. Indeed, the higher the percentage of iron in the sample, the greater the observed shift. Moreover, in each sample, a peak at 288.6–289.0 eV was observed and can be referred to as Ti–O–C = O (Xing et al. 2014) or the presence of C = O species (Xing et al. 2014; Roy et al. 2019; Wtulich et al. 2022). Finally, it was noteworthy to observe that only TiO_2__SCS350 and P25 samples exhibited a further peak localized at 286.8 eV, which could be due to the presence of an ether group C–O–C (Kavitha and Devi 2014; Xing et al. 2014; Sivaranjini et al. 2018) that was more evident in the commercial sample. Its presence could be due to the procedure adopted and it was especially true for the sample obtained through SCS synthesis (Lopera et al. 2018). Finally, Fig. 4**d** reports the deconvolution of Fe *2p* spectra for Fe/TiO_2__SCS and Fe/TiO_2__HT. Both samples exhibited peaks associated with the presence of Fe^2+^ and Fe^3+^ species at a binding energy of 709.0 eV and 711.3 eV for Fe/TiO_2__SCS and 708.8 eV and 712.6 eV for Fe/TiO_2__HT (Freyria et al. 2017; Hou et al. 2019; Gao et al. 2020; Huang et al. 2023). The satellite at 717 eV further confirmed the presence of Fe^3+^ and Fe–O–Ti in the structure (Bharti et al. 2016). Surprisingly, the Fe/TiO_2__SCS sample exhibited a further peak that can be ascribed to the presence of metallic iron Fe^0^, with the peak centered at a binding energy of 707.3 eV (Hou et al. 2019). This finding suggests that, compared to the hydrothermal technique, the SCS method is capable of obtaining metallic iron on the surface, which may have positive effects on titania performance. Moreover, considering the amount of all the species, in both cases, the percentage of Fe^3+^ was almost the same, whereas Fe^2+^ was extremely different, 60.5% in Fe/TiO_2__SCS and 76.4% in Fe/TiO_2__HT, due to the presence of Fe^0^ (17.3%) in the former.

**Fig. 5:**
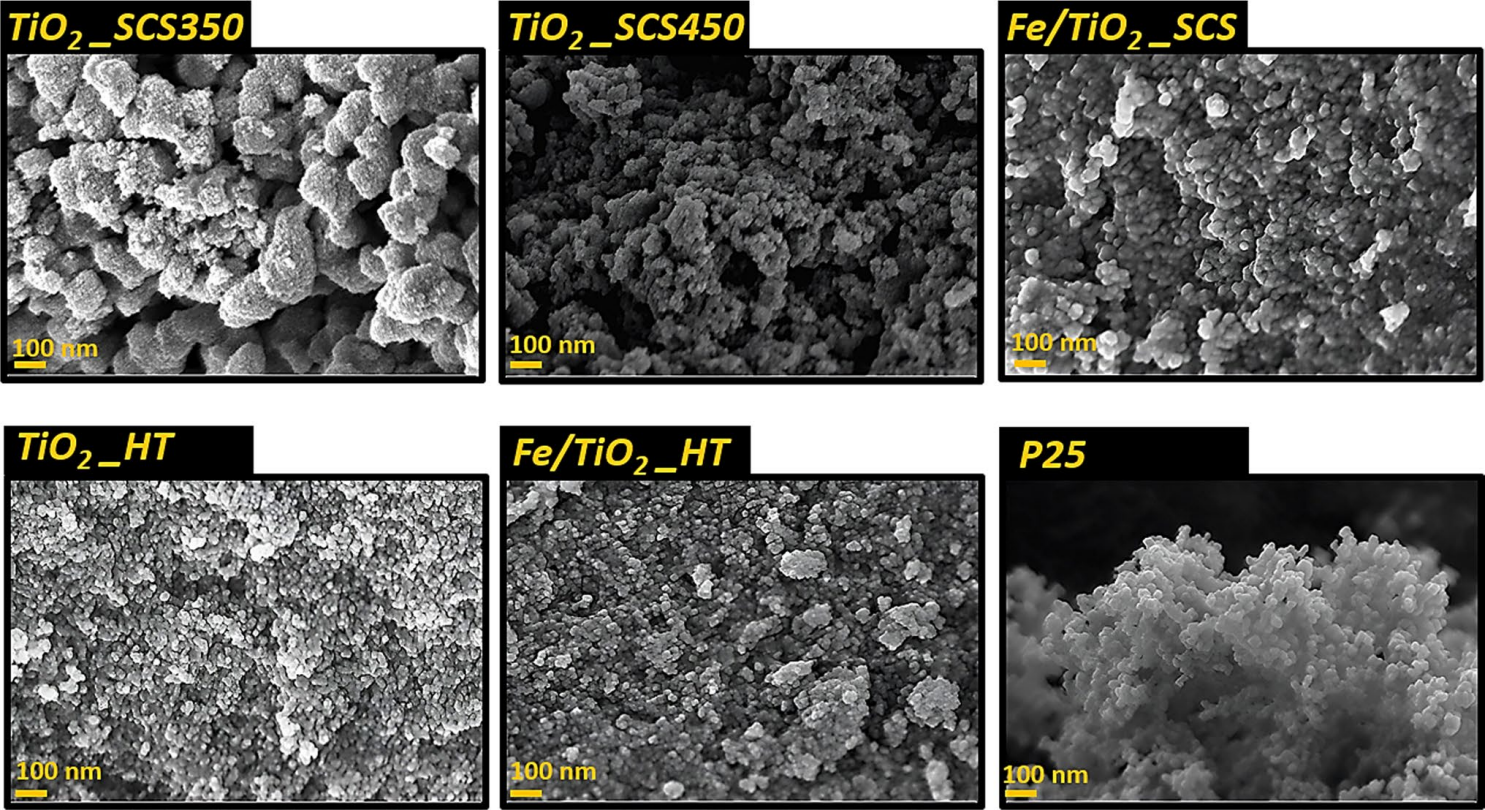
FESEM images of the different mesoporous titania samples

FESEM analysis was used to examine the surface morphology of the samples, as reported in Fig. 5.

**Fig. 6:**
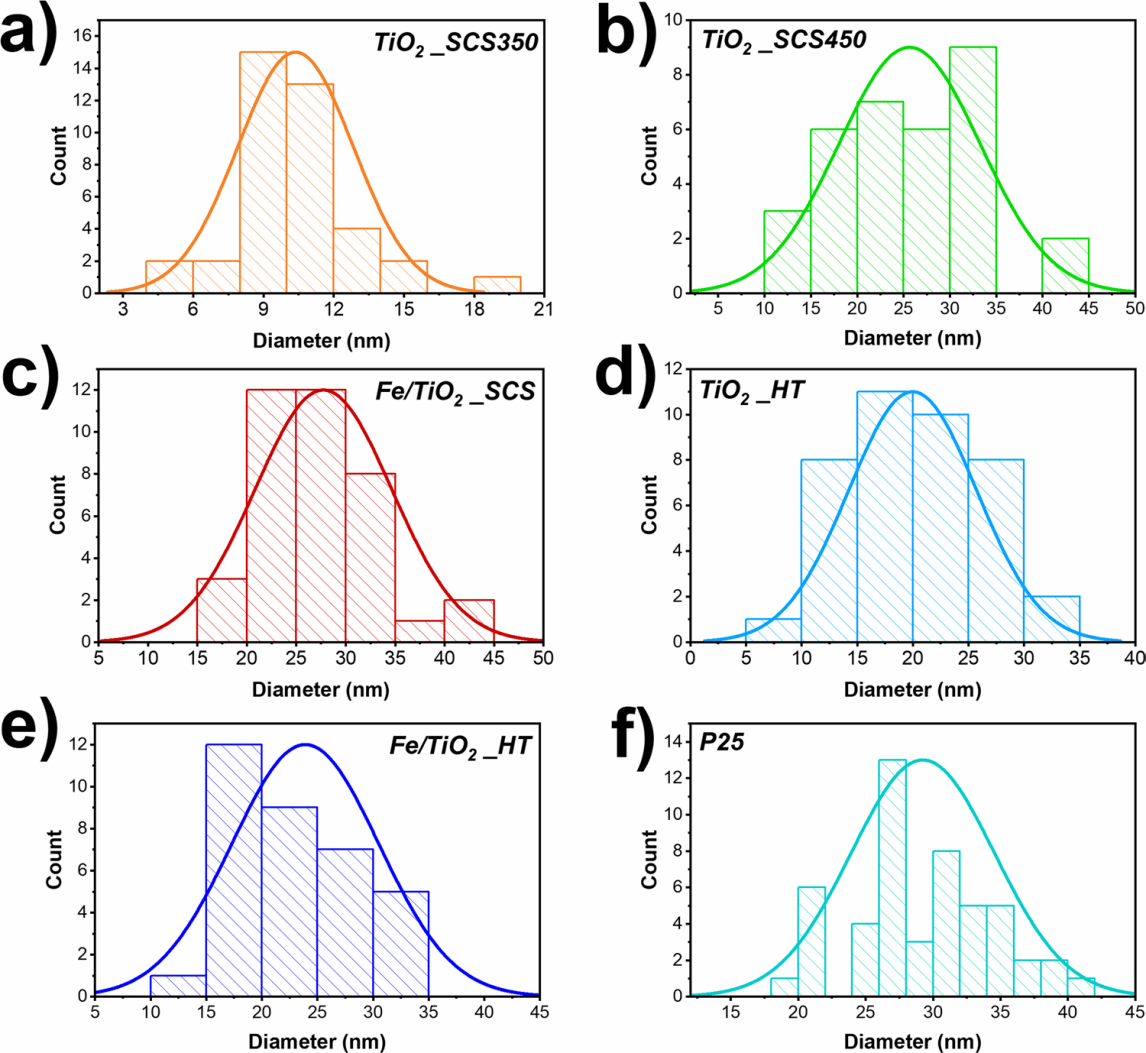
Particle size distributions of TiO_2__SCS350 (**a**), TiO_2__SCS450 (**b**), Fe/TiO_2__SCS (**c**), TiO_2__HT (**d**), Fe/TiO_2__HT (**e**), and P25 (**f**) evaluated with Image J software

In all the samples investigated, the presence of spherical nanoparticles with a size of a few tens of nanometers can be seen, as found in other works (Ba-Abbad et al. 2012; Di Carlo et al. 2013; Freyria et al. 2017; Blangetti et al. 2023; Mancuso et al. 2023). Furthermore, in contrast to the other samples, TiO_2__SCS350 exhibited a morphology made of large compact aggregates (ca. 200 nm) that almost resemble the appearance of a sponge, composed of small spherical nanoparticles and a porous structure. In addition, it was interesting to note that the particle size distribution varied according to synthesis temperature and procedure (see Fig. 6).

With the same synthesis procedure, mainly SCS, a Gaussian curve was observed to be more spread out at higher temperatures, i.e., in the case of TiO_2__SCS450, with a particle size distribution in the range of 10–45 nm and an average size of approx. 25 nm. By decreasing the synthesis temperature (thus in the case of TiO_2__SCS350), a decrease in particle size was observed, with an average value of 11 nm. By comparing this value with that of the crystallite size obtained from the XRD analysis by applying the Scherrer equation, it can be concluded that a particle of TiO_2__SCS350 was a single crystal, just as in the case of P25. On the other hand, in Fe/TiO_2__SCS, it can be seen that the average size has shifted towards higher values, showing the presence of particles consisting of several crystallites. The increase in size due to the presence of a heteroatom in the structure has also been found in the literature (Mozammel and Jalali 2017). A similar reasoning applied to TiO_2__HT and Fe/TiO_2__HT samples.

**Fig. 7:**
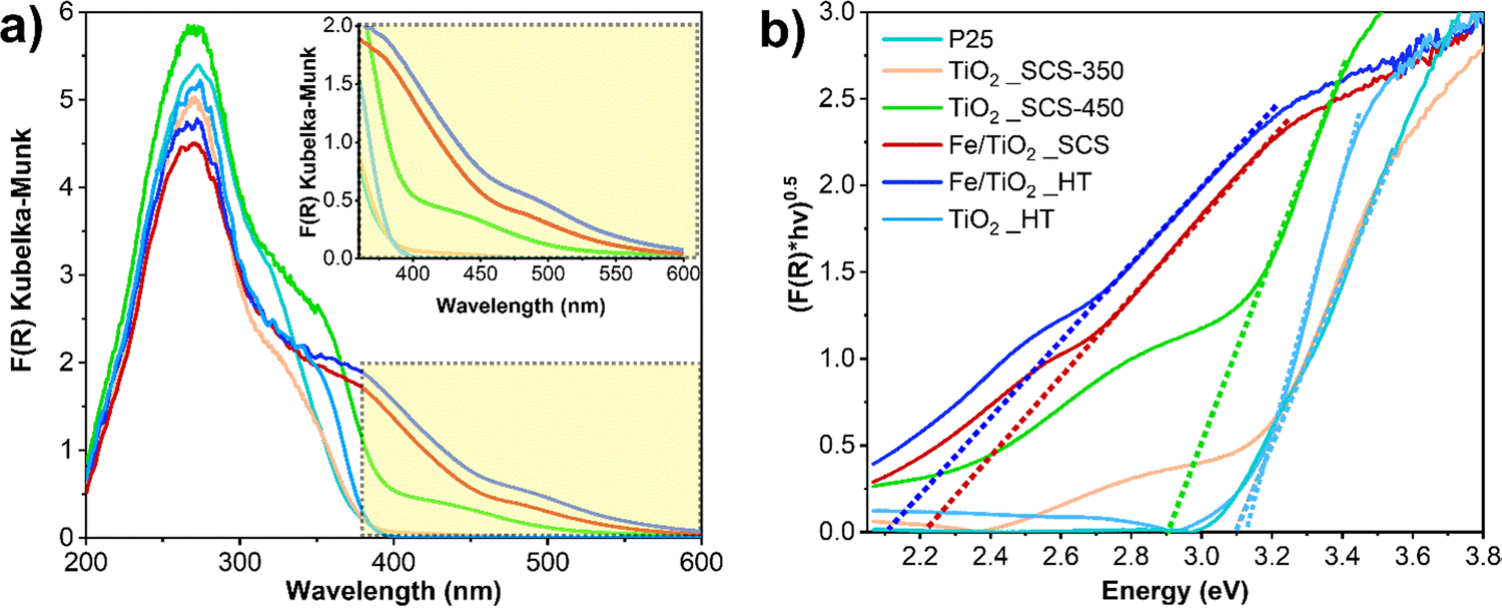
DR UV–Vis spectra of the investigated samples (**a**) and corresponding Tauc’s plot, obtained by assuming a behavior of indirect semiconductors (**b**)

Finally, by processing the results obtained from DR-UV–Vis spectroscopy, the optical band gap (*E*_*g*_) value can be easily determined from absorption spectra and Tauc’s Plot (see Fig. 7). It is well known that, depending on the crystalline phase present in the structure, a direct (i.e., for the Rutile and Brookite) or indirect (i.e., for Anatase) band gap should be considered. For simplicity, since all the samples exhibited the presence of anatase as a major crystalline phase, only the indirect band gap was reported.

By comparing the Kubelka–Munk function (F(R)) for all the samples investigated (see Fig. 7**a**), it appeared clear that the synthesis procedure and the calcination temperature adopted to obtain the catalysts as well as the presence of iron played a crucial role. Precisely, it was interesting to observe that in the region between 350 and 370 nm, the spectra related to P25 and TiO_2__SCS350 were overlapping, showing similar slopes. In fact, by evaluating the *E*_*g*_ from the Tauc’s Plot (Fig. 7**b**), both samples exhibited the same value equal to 3.08 eV. On the other hand, TiO_2__HT and TiO_2__SCS450 presented more pronounced slopes. It was interesting to highlight the role of both synthesis procedures and temperatures adopted. By comparing the samples obtained through the SCS method, the TiO_2__SCS450 sample extended its absorption towards longer wavelengths, above 400 nm. This region could be associated with the Urbach tail, which is namely an exponential part in the energy spectrum of the absorption coefficient that may appear near the optical band edge in amorphous, disordered, and crystalline materials (Gervasi et al. 2023). This region could be related to the presence of disorder and physisorbed water (see OH species concentration from XPS results), or was due to the higher temperature adopted during the synthesis procedure, since it was demonstrated that this parameter affects the Urbach tail (Aljishi et al. 1990; Yap et al. 2020). This phenomenon in TiO_2__SCS450 provoked a decrease in the *E*_*g*_, resulting in 2.89 eV. On the contrary, TiO_2__HT did not show an Urbach tail, thus exhibiting a slightly higher value of *E*_*g*_ (3.13 eV) compared to both TiO_2__SCS350 and TiO_2__SCS450. Finally, by considering both the iron-containing samples, it was observed a more pronounced red-shift. In these cases, the phenomenon was due to the presence of iron in the structures, which helps to decrease the band gap thanks to the presence of defects and produce an absorption tail extending deep into the forbidden gap, as reported in the literature (Sharma et al. 2017; Choudhury and Choudhury 2014). Moreover, introducing Fe could also increase the efficient heterojunctions formed at the interfaces, which promoted the separation of electron–hole pairs and transferred them to the catalyst surface for the degradation reaction (Yap et al. 2020; Gadhi et al. 2023). Furthermore, it is important to remember that the greater the extent of the Urbach tail, the smaller the band gap. From Tauc’s plot, Fe/TiO_2__HT exhibited lower *E*_*g*_ compared to Fe/TiO_2__SCS (2.11 and 2.22 eV respectively). This was consistent with the EDX analysis since the former had a higher Fe content compared to the latter.

### Photocatalytic activity

**Fig. 8:**
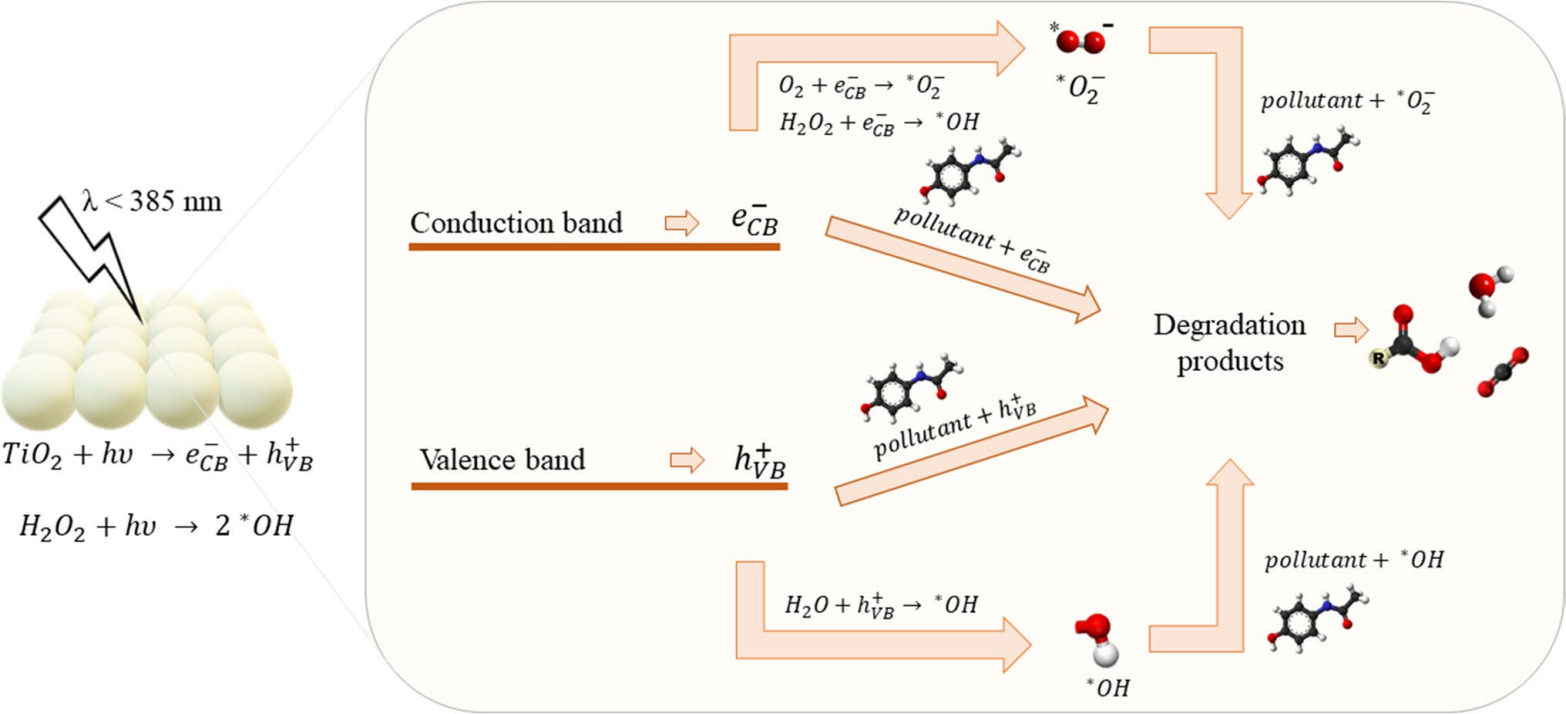
Simplified scheme of activation of TiO_2_ in the presence of a light source for the degradation of paracetamol

**Fig. 9:**
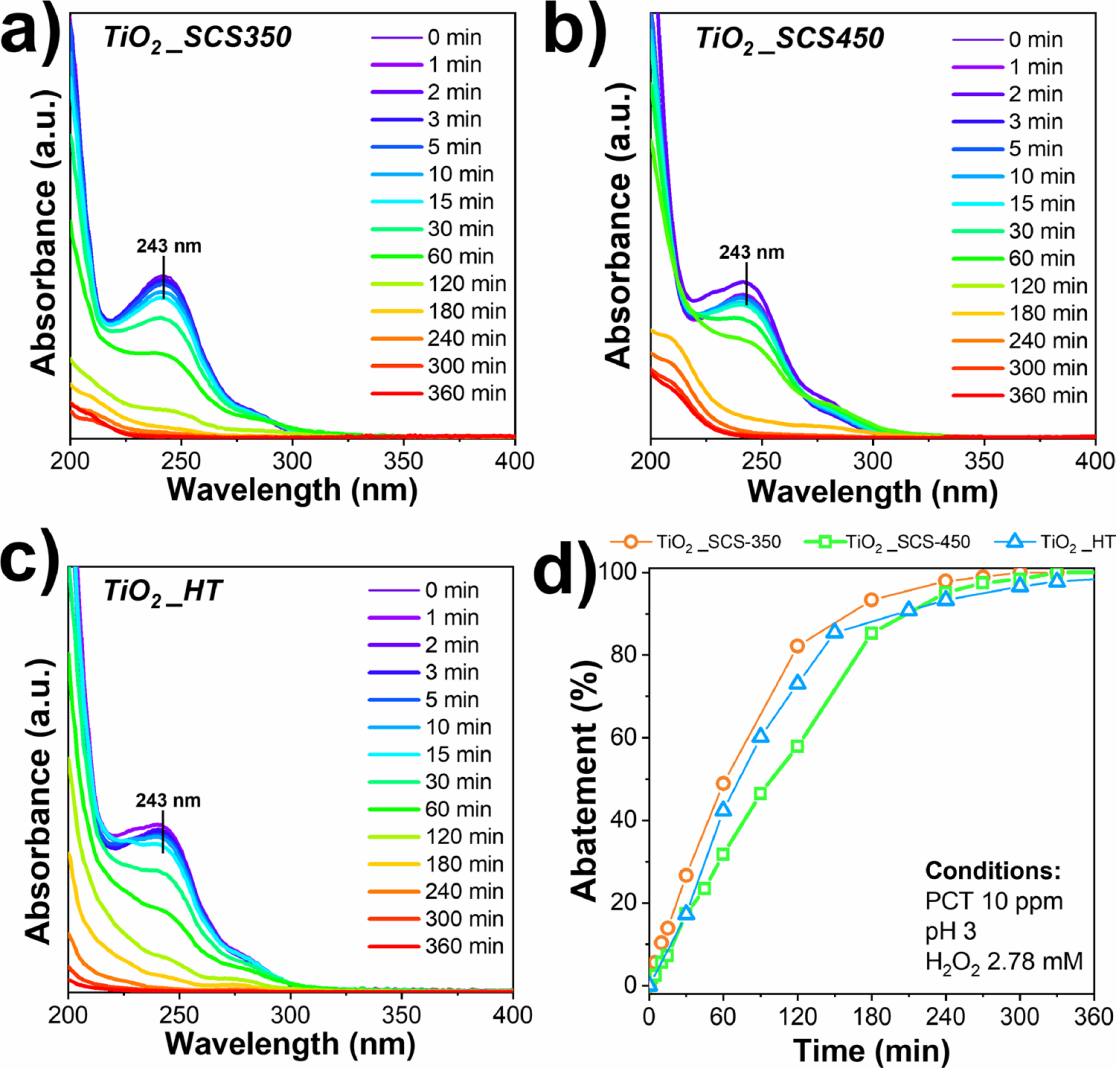
Paracetamol degradation spectra on TiO_2__SCS350 (**a**), TiO_2__SCS450 (**b**), and TiO_2__HT (**c**) under UV light source (302 nm), initial concentration of paracetamol = 10 ppm, catalyst dosage = 2 g L^−^^1^, pH 3, H_2_O_2_ 2.78 mM, temperature of 25 °C. Abatement of pollutant (**d**) as a function of the time on the mesoporous TiO_2_

Before starting, preliminary tests were carried out to optimize the amount of H_2_O_2_ to be used in the degradation of paracetamol (see Fig. S4), to avoid problems related to band overlap between H_2_O_2_ and the pollutant and to avoid ·OH radicals reacting preferentially with H_2_O_2_ rather than with paracetamol (Samira et al. 2022). For this reason, the amount of hydrogen peroxide was set at 2.78 mM and the same value was maintained in each test. The results of the catalytic degradation of paracetamol in dark conditions were reported in Fig S5. From the literature, the characteristic peak for the evaluation of paracetamol degradation is the one located at 243 nm (Trujillano et al. 2022). As expected, no degradation occurred in this condition since all the samples were not active in the absence of light, due to the lack of reactive species that should be generated thanks to the interaction with the light source. Since no lowering of the characteristic peak of paracetamol (λ = 243 nm) was recorded and no new peaks concerning the presence of any by-products were identified, the test was stopped after 2 h for all the samples investigated. This evidence could suggest that the paracetamol exhibits a stereochemical configuration which is unsuitable to chelate with titania, leading to negligible chemisorption on catalysts’ surface (Yang et al. 2008). When the catalyst was irradiated by UV light, the degradation of the pollutant started thanks to the activation of the material. In fact, when the TiO_2_ was irradiated with energy higher than its band gap one, valence band holes and conduction band electrons were generated and subsequently they can directly react with pollutant adsorbed on the catalyst surface or generate radicals (i.e., ·OH, ·O_2_^−^) which in turn react with pollutant (Yang et al. 2008; Moctezuma et al. 2012; Freyria et al. 2018). Moreover, the presence of H_2_O_2_ plays an important role since it can also interact with the irradiation, producing further radicals that can enhance the degradation of pollutants. A simplified scheme is proposed in Fig. 8, whereas the spectra for the degradation of paracetamol on the pure TiO_2_ samples are shown in Fig. 9.

By comparing the pristine samples, it was evident the effect of the different synthesis procedures adopted, which affected the physico-chemical properties, and consequently, the catalytic performance of the samples, as shown in Fig. 9. In each case, it can be noted that paracetamol achieved complete degradation, but the TiO_2__SCS350 sample turned out to be the best, reaching 100% abatement after 300 min, while the other two catalysts achieved the same result at times longer than 360 min. More specifically, as the reaction proceeded, a change in the profile of the spectra was observed after 15 min. It is important to remember that paracetamol has two characteristic peaks, positioned at wavelengths of 190 nm and 243 nm, and both decreased in intensity during the reaction, until they disappeared, thus indicating the degradation of the pollutant. Analyzing the spectra obtained during the reaction, it was interesting to note several peaks attributable to the formation of different intermediates. In particular, the peak at 208 nm, which is most visible for the TiO_2__SCS450 sample, may indicate the formation of 1,2,4-trihydroxybenzene molecule, whose presence was noted approximately 30 min after starting the test. On the other hand, the second band located at around 290 nm could indicate the formation of the hydroquinone intermediate, which was subsequently degraded after 180 min in the case of TiO_2__SCS350, and after 240 min in the case of both TiO_2__HT and TiO_2__SCS450. Finally, a lower intensity band at around 315 nm ascribable to the formation of 4-nitrophenol was detected after 30 min but was rapidly degraded in all cases tested. Such by-products/intermediates were also detected in several works in the literature (Moctezuma et al. 2012; Blangetti et al. 2023).

**Fig. 10:**
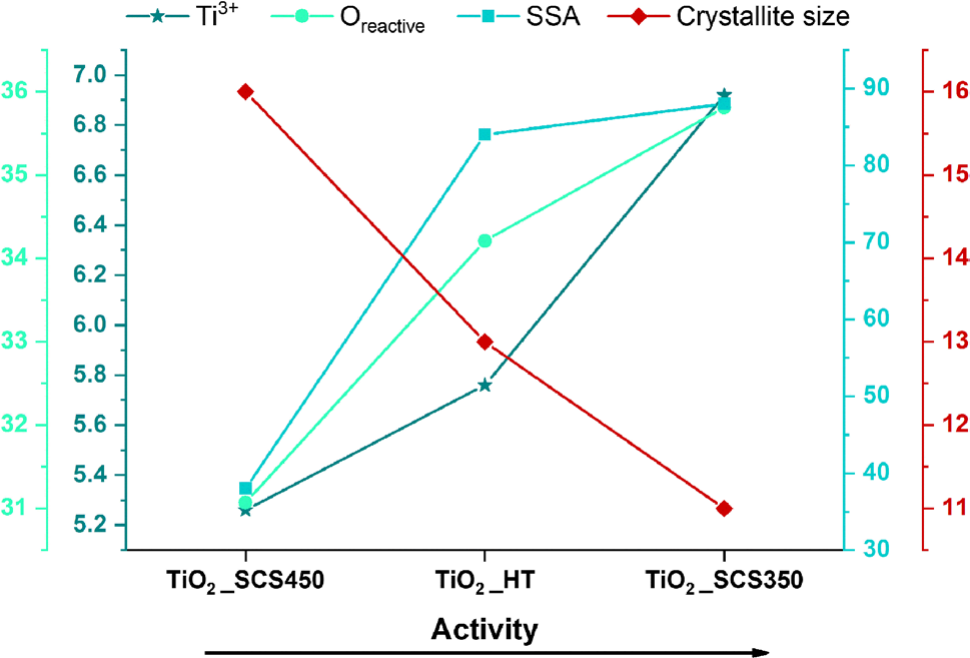
Correlation between the catalytic performance of TiO_2__SCS350, TiO_2__SCS450, and TiO_2__HT and their physico-chemical properties for the degradation of 10 ppm of paracetamol under UV light source, pH 3, and temperature of 25 °C

Therefore, from the results, it was interesting to note how both the synthesis procedure and the temperature influenced the properties of the samples. In particular, the results were in perfect agreement with the findings of the physico-chemical characterizations. Specifically, from the XPS analyses, a higher content of Ti^3+^ species and defective oxygens (O_defective_) was found in the TiO_2__SCS350 sample (see Table 3), which can be attributed to surface defects that had a positive effect on catalytic performance. In addition, the N_2_-physisorption results and XRD analysis showed the highest surface area (88 m^2^g^−1^) and lowest crystallite size (5 nm) for the same sample, respectively, which are crucial parameters (Freyria et al. 2018). As the Ti^3+^ and O_react_ species decrease from one sample to the next, a progressive decrease in removal kinetics can be seen (in the order TiO_2__SCS350 > TiO_2__HT > TiO_2__SCS450). From these considerations, the SCS method at low temperatures (mainly 350 °C) led to a more defective structure in terms of Ti^3+^ species and reactive oxygen species, as well as the highest specific surface area and lowest crystallite size (see Table 1 and Table 2). Figure 10 reports the catalytic activity as a function of the results of the chemical-physical characterizations. In particular, the TiO_2__SCS350 sample, having a larger surface area than the other two samples, may have a greater number of active sites per unit area, and contribute a greater amount of defective species, which may therefore increase catalytic performance (Piumetti 2022). Therefore, it was clear that the synthesis conditions play a key role in determining catalytic performance.

In addition, to study the stability of TiO_2__SCS350, some physico-chemical characterizations were carried out on the spent catalyst after its recovery at the end of the reaction. For the sake of brevity, the results of the XRD and XPS analyses are reported in the Supporting Information section (see Fig. S6 and S7). Overall, exposure to the UV light source and the reagent system for the duration of the test did not cause any changes in the crystal structure, as detected by XRD. On the other hand, when comparing the deconvoluted spectra of C 1 s, O 1 s, and Ti 2p, certain differences between the fresh and spent catalysts were revealed. In particular, the presence of Ti^2+^ species was no longer detected and the amount of defective oxygen (O_α_) decreased slightly (from 25.0 to 15.8%), probably due to its active role during the reaction.

**Fig. 11:**
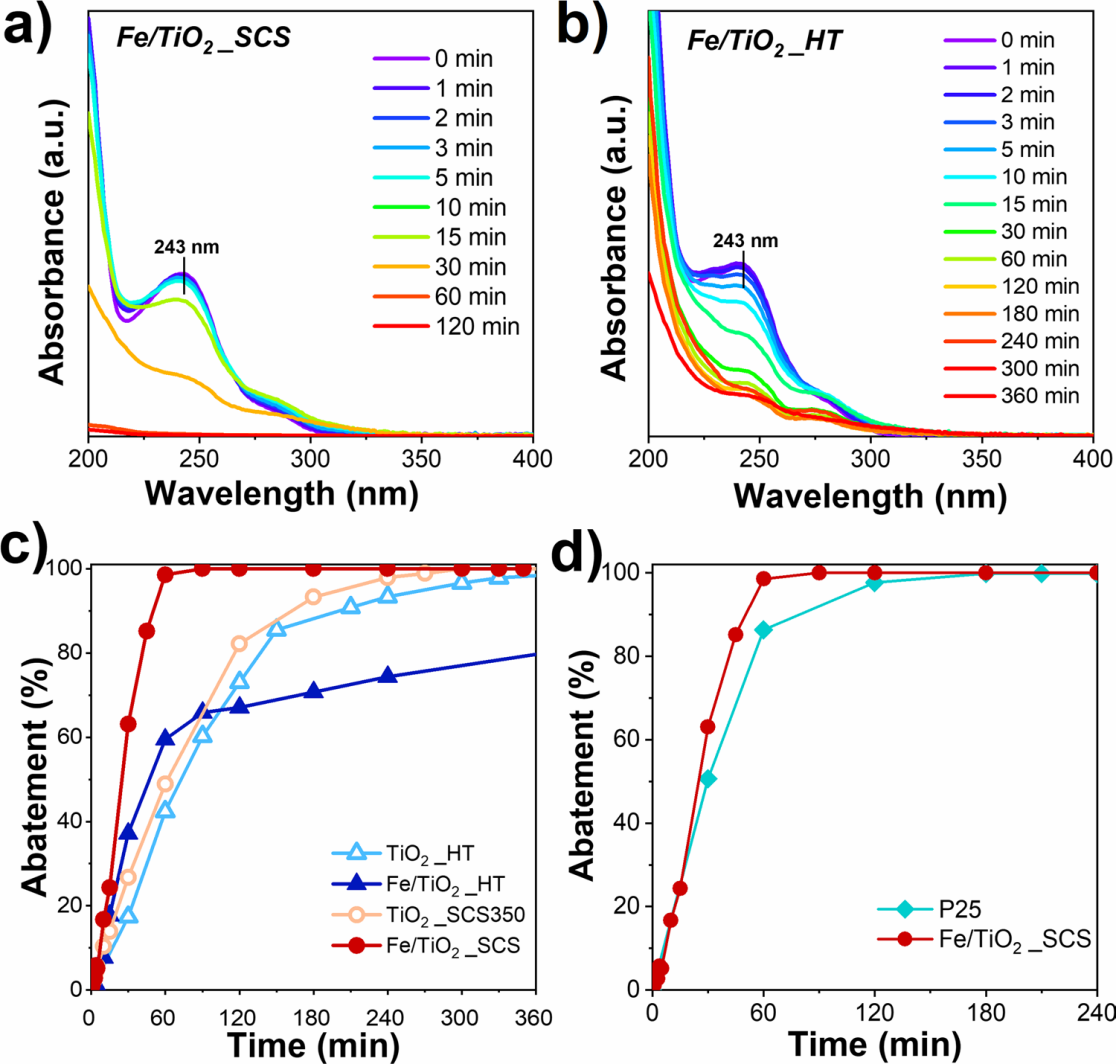
Paracetamol degradation spectra on Fe/TiO_2__SCS (**a**), Fe/TiO_2__HT (**b**) under UV light source (302 nm), initial concentration of paracetamol = 10 ppm, catalyst dosage = 2 g L^−^^1^, pH 3, H_2_O_2_ 2.78 mM, temperature of 25 °C. Comparison of catalytic performance (abatement vs. time) between pure and Fe-containing samples (**c**) and between Fe/TiO_2__SCS and P25 (**d**)

The catalysts with the best performances, mainly TiO_2__SCS350 and TiO_2__HT, were subsequently modified with iron, and the respective Fe-containing samples, named Fe/TiO_2__SCS and Fe/TiO_2__HT were tested for the same purpose and in the same conditions in order to investigate the influence of the metal in the structure. The results are reported in Fig. 11.

As a whole, the presence of iron was advantageous for the degradation of the pollutant, since was able to enhance and increase the kinetic of removal. Precisely, Fe/TiO_2__SCS exhibited outstanding performance compared to the pure samples and other studies in the literature (Yap et al. 2020). Observing the spectra shown in Fig. 11 for Fe/TiO_2__SCS (**a**) and Fe/TiO_2__HT (**b**), the same peaks were found as in Fig. 9 for the intermediates 1,2,4-trihydroxybenzene (peak at 208 nm) and hydroquinone (peak at 290 nm). Interestingly, with the Fe-containing materials, a further band was found at around 320 nm, attributable to p-nitrophenol (Moctezuma et al. 2012), which was not detected with the pristine ones and in P25 spectra (see Fig. S8). Surprisingly, Fe/TiO_2__SCS was able to completely degrade paracetamol (whose peak is located at 243 nm) as well as all the reaction intermediates in about 60 min, reaching 100% abatement, unlike the Fe/TiO_2__HT sample which, after 60 min, had reached just under 60% conversion, and after 360 min there was still the presence of both paracetamol and the various reaction intermediates. The results showed that the iron brought an improvement in catalytic performance over the pure samples (Fig. 11**c**), and again, the synthesis procedure proved to be a crucial point. In fact, it was interesting to note that with the SCS method, it was possible to obtain a higher surface area and smaller crystallite size, respectively (see Table 1 and Table 2), although the difference with the HT sample was not so great. Another important point that affected the performance was the nature and the amount of iron species. Precisely, the presence of Fe^0^ nanoparticles was detected in Fe/TiO_2__SCS, which were not present in the sample obtained by hydrothermal technique, as demonstrated by the XPS spectra (see Fig. 4**d**), and therefore may have increased the catalytic activity. Indeed, metal nanoparticles on the TiO_2_ surface can prevent electron–hole recombination and readily transfer electrons to oxygen or water molecules to form hydroxyl radicals for the enhancement of the photocatalytic activity (Cifci et al. 2017). Moreover, to explain the difference between the two iron-containing samples, it is necessary to also consider the amount of a certain species. For example, in another work, it was demonstrated that an excess of Fe^2+^ species could reduce the efficiency of the degradation process, due to undesirable reactions between the hydroxyl radicals and the ions of ferrous iron, that produce Fe^3+^ which are not suitable for the degradation (Samira et al. 2022). Thus, the high amount of Fe^2+^ species in Fe/TiO_2__HT may have caused a sort of inhibition, decreasing performance compared to the Fe/TiO_2__SCS sample. Finally, in Fig. 11**d**, the comparison between P25 and the most-performing synthesized catalyst was reported. It was noteworthy that the presence of both anatase and rutile in the commercial sample P25 allowed superior photocatalytic performance, due to the stabilization of electron/hole pairs, lowering their recombination (Freyria et al. 2017; Freyria et al. 2018). However, even in this case, Fe/TiO_2__SCS was shown to have better catalytic activity due to the presence of iron, confirming the effectiveness of the SCS technique, which is also less impactful than the technique used to obtain P25 (generally pyrolysis at temperatures higher than 1800 °C), as reported in the literature (Manzoli et al. 2022). Moreover, the presence of iron could solve an important disadvantage related to the difficulty of separating titania powder after the decontamination (Borges et al. 2015), since it was demonstrated the presence of a magnetic phase that could ensure easy separation of solids after use, making it more attractive for practical use (Freyria et al. 2017).

Since, Fe/TiO_2__SCS proved to be the best catalyst, post-reaction characterizations, mainly XRD, XPS, and ICP analyses. In particular, the spent catalyst was monitored during the entire reaction time, specifically after 5 min (which corresponded to the time when the paracetamol conversion started), 30 min (which was half of the reaction time), and finally 60 min (which corresponded to the end of the reaction, when the paracetamol conversion was 100%). For the sake of brevity, the results are reported in the Supporting Information section. XRD diffractograms (Fig. 9) showed no changes in the crystal structure during the entire reaction time. Furthermore, analyzing the solution after 5 min, 30 min, and 60 min, the ICP analysis revealed that the Fe concentration gradually increased (from 4.3 to 5.1 ppb); however, iron leaching was negligible, indicating that the concentration of dissolved Fe is not the main factor involved or that iron reacts immediately with H_2_O_2_. Moreover, this fact is positive as, by preventing iron leaching, water contamination is avoided. Finally, it was interesting to observe how the iron species vary during the reaction on the catalyst surface. Indeed, an oscillating behavior involved some species on the catalyst surface, mainly Fe^2+^ and Fe^3+^ species, as detected from XPS results (Fig. S10). It was interesting to note that at the beginning of the reaction (after 5 min), a strong increase in Ti^2+^ species was detected, reaching 73%. The formation of this species resulted from the reduction of Fe^3+^ and is required to initiate the Photo Fenton process (Minella et al. 2014; Xu et al. 2019). As the reaction proceeded, both species oscillated until, in the end, the concentration of Fe^2+^ decreased and Fe^3+^ gradually increased, reaching almost the initial percentage, as evidenced by the deconvolution of the XPS spectra. This phenomenon could be due to the redox cycle of Fe causing regeneration of initial Fe species. This could be the reason why, after 60 min, the concentration of Fe^2+^ began to decrease and, as a result, the concentration of Fe^3+^ species increased slightly. Therefore, these outcomes suggest that the presence of iron on the surface of titania leads to the in situ formation of species that combine photocatalysis and photo-Fenton in one system (Puri et al. 2021).

Finally, it may be of interest to perform a comparison between the best-performing catalysts and the performance of other catalysts present in the literature, by considering similar operation conditions. Yap et al. (2020) adopted the sol–gel procedure to obtain titania, used as a catalyst for the removal of paracetamol (5 mgL^-1^, catalyst dosage = 1.0 g L^-1^), but only 30% conversion was obtained after 2 h. In other works, to improve performance, titania was supported on hydrotalcite (Conference et al. 2023) or zeolite (Jayasree and Remya 2022) respectively reaching 40% after 3 h (10 mg L^-1^, catalyst dosage of 3.0 g L^-1^) and 96% after 90 min (10 mg L^-1^, catalyst dosage of 2.0 g L^-1^). Other works reported the presence of other species to enhance the catalytic activity. For instance, Namshah et al. (Namshah and Mohamed 2018) synthesized TiO_2_ containing 3 wt% of WO_3_ which was able to abate 50 ppm in 60 min with 1 g L^-1^ of catalyst dose. Furthermore, Puri et al. (2021) employed a dip-coating method to produce Fe/TiO_2_ composite and, subsequently, tested to remove 10 mg L^-1^ of paracetamol by introducing 525 mg L^-1^ of H_2_O_2_. The results showed that a complete degradation of the pollutant occurred after 250 min. In another work, Vaiano et al. (2018) synthesized a graphite titania composite (3 g L^-1^) to remove paracetamol at different concentrations. Again, the results showed that in the case of an initial concentration of 12.5 ppm and catalyst dose of 3 g L^-1^, the catalyst was able to remove the pollutant after 180 min. Finally, Yap et al. (2020) reported the use of Fe/TiO_2_ containing 3wt% of Fe to abate 5 ppm of paracetamol with a catalyst dose of 1 g L^-1^. The results showed that a pollutant degradation of 70% was achieved after 2 h, without reaching a complete removal. All these examples demonstrate that the results obtained in this work with catalysts synthesized via SCS perform similarly to and/or better than those found in the literature. Therefore, this highlights that the SCS technique represents a viable alternative to conventional synthesis techniques, being environmentally friendly, low-cost, and non-time-consuming.

### Kinetic results

To better investigate the performance of all samples, a kinetic study was performed and the results are reported in Table 4.

**Table 4:** Kinetic parameters for the degradation of paracetamol over all the samples investigated

Kinetic model	Parameters	TiO_2__SCS350	TiO_2__SCS450	TiO_2__HT	Fe/TiO_2__SCS	Fe/TiO_2__HT	P25
	*C*_0,exp_ [mg L^−1^]	10.3659	9.004	10.5007	11.3289	10.3111	9.0519
	*t*_1/2,exp_ [min]	67	99	72	25	47	30
PFO	*R*^2^	0.9943	0.9784	0.9451	0.9950	0.8761	0.9619
	*k*_1_ [min^−1^]	0.0110	0.0055	0.0078	0.0191	0.0145	0.0174
	*C*_0,fit_ [mg L^−1^]	10.3181	8.9665	11.8675	11.4524	10.6451	9.0784
	*t*_1/2,PFO_ [min]	63	126	89	36	48	40
PSO	*R*^2^	0.9961	0.9802	0.9489	0.9921	0.8645	0.9670
	*k*_2_ [L mg^−1^ min^−1^]	0.0012	0.0006	0.0007	0.0019	0.0016	0.0021
	*C*_0,fit_ [mg L^−1^]	10.3306	8.9686	11.8765	11.5074	10.6952	9.0992
	*t*_1/2,PSO_ [min]	81	186	120	46	58	52

Comparing the determination coefficient value (*R*^2^) values obtained by fitting the experimental data with a pseudo-first-order (PFO) and pseudo-second-order (PSO) model, it can be seen that these results were quite similar to each other and were quite close to the unity in all cases. Thus, to better discriminate the accuracy of the model that best fits the data, it was necessary to consider the kinetic parameters obtained, in particular the initial concentration (*C*_0_) and the half-life (*t*_1/2_), which were compared with the experimental ones. Moreover, in all cases, a substantial difference of the kinetic constants *k*_1_ and *k*_2_ was observed, since the latter was an order of magnitude less than the former. This was consistent with the kinetic theory. In fact, it is well known that in a PSO model (in which the reaction rate depends on the square of the reactant concentration), it is necessary to have a collision between two molecules for the reaction to occur. The probability that this can happen is lower than in a PFO model, in which only one molecule is required and in which the rate of a PFO reaction depends on the first power of the reactant concentration. Therefore, the rate of a second-order reaction proceeds more slowly than that of a first-order reaction. The results obtained are therefore in agreement with the theory.

**Fig. 12:**
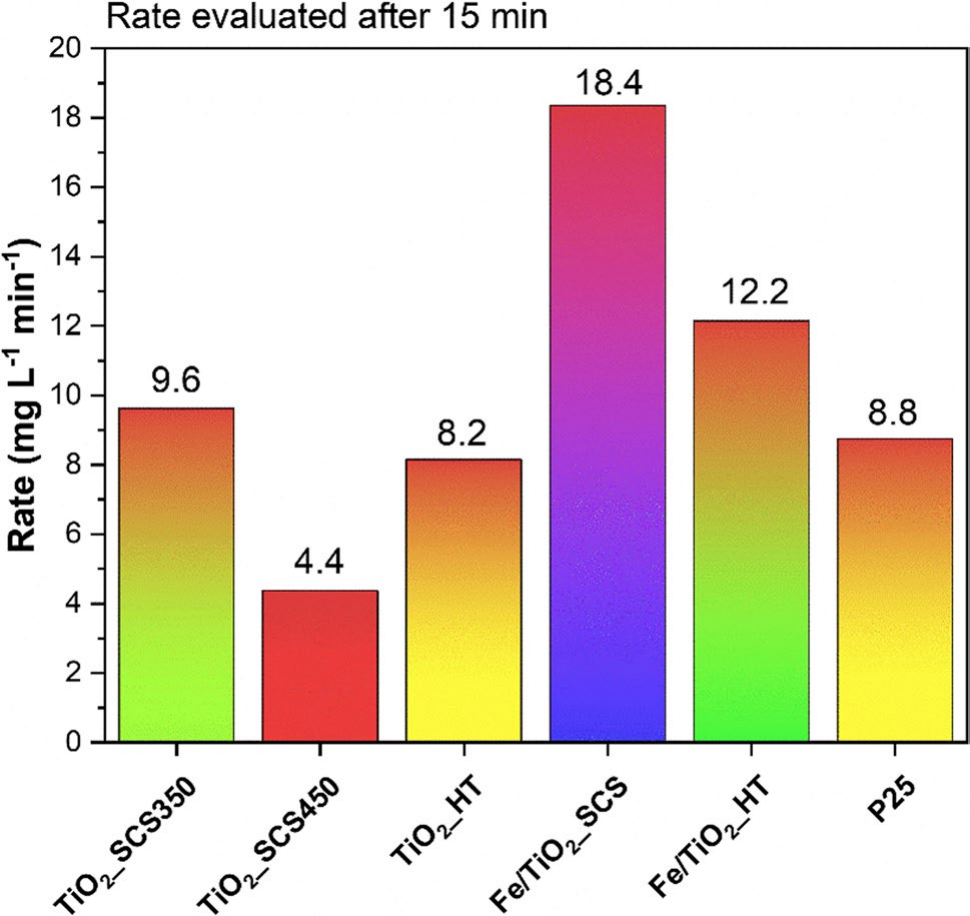
Conversion rate of all investigated samples evaluated in the kinetic regime at a reaction time of 15 min

Concerning the most suitable model for describing the experimental data, for almost all the cases analyzed, although the PSO determination coefficient *R*^2^ was slightly higher, the PFO model was found to be the best, especially concerning the half-life, which, with this model, was closer to the real one. This was also consistent with other studies (Yang et al. 2008; Audino et al. 2019; Macías-García et al. 2019; Zanchettin et al. 2021; Rajamehala et al. 2022; Blangetti et al. 2023) which reported that PFO is the most suitable to describe the degradation of paracetamol. Finally, the degradation rate of the pollutant was evaluated in the kinetic regime (conversion not higher than 20%) and the results are reported in Fig. 12. As highlighted, the Fe samples exhibited the highest rates equal to 18.4 and 12.2 mg L^−1^ min^−1^, for Fe/TiO_2__SCS and Fe/TiO_2__HT, respectively, whereas, among the bare samples, TiO_2__SCS350 proved to be the best, with a rate of 9.6 mg L^−1^ min^−1^ in 15 min. Taking into consideration the results obtained, it was possible to affirm the effectiveness of the SCS method for the synthesis of high-performance materials for the degradation of paracetamol.

### Role of pH and H_2_O_2_ on the best-performing catalysts

**Fig. 13:**
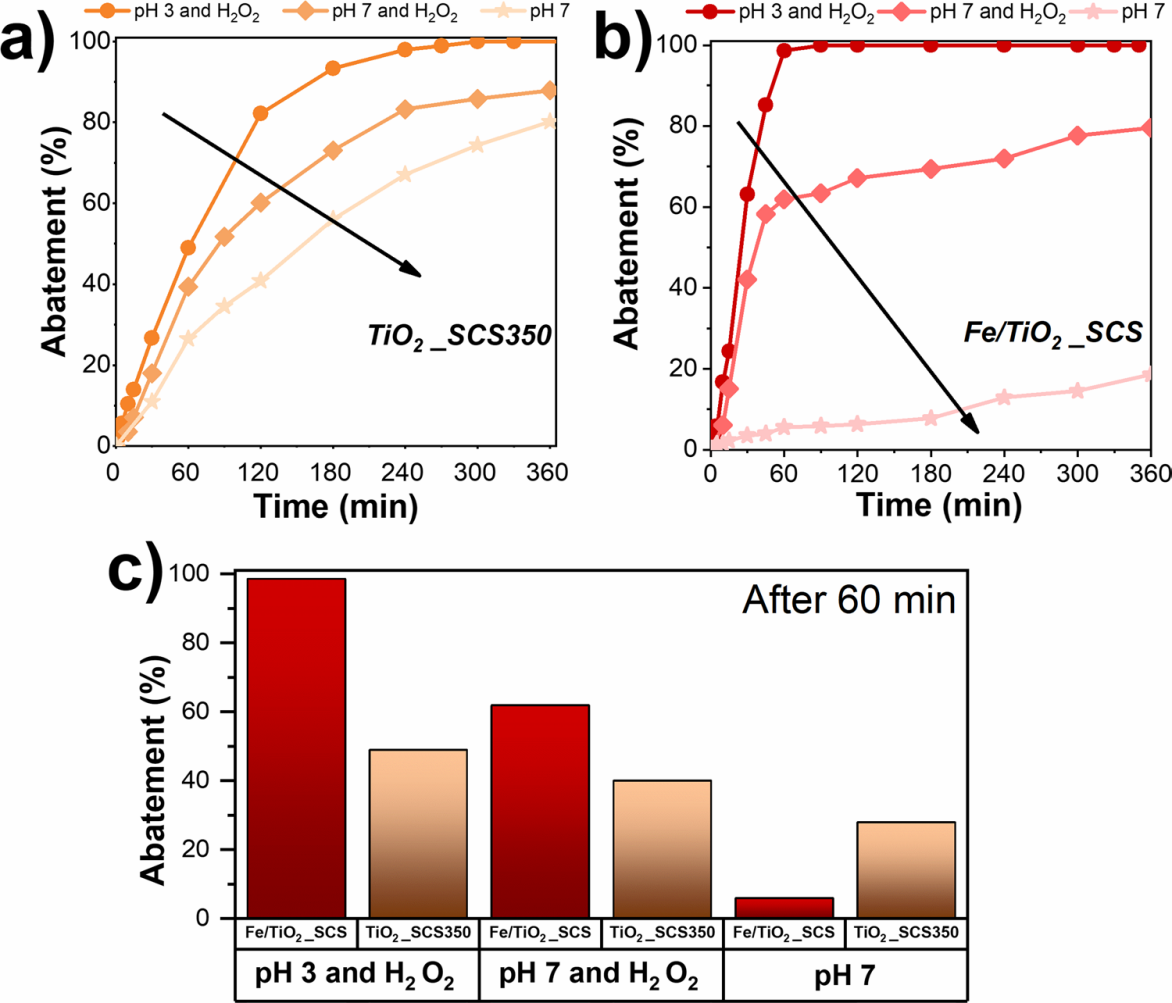
Variation of paracetamol abatement over time at different operating conditions for TiO_2__SCS350 (**a**) and Fe/TiO_2__SCS (**b**). Comparison of catalytic performance after a reaction time of 60 min for the two catalysts

To further investigate the performance of the SCS-synthesized catalysts and confirm their effectiveness in removing paracetamol, additional tests were conducted by varying the pH and excluding the presence of H_2_O_2_. The results are shown in Fig. 13.

As a whole, by observing the abatement over time, for both catalysts, it can be seen that the best operating conditions were those for which there was an acidic pH and the presence of H_2_O_2_. This is in agreement with the literature (Van et al. 2020). When the pH increased, a worsening of performance occurred; in particular, a decrease in the slope of the curve was observed, thus attributable to a slowing of the kinetics. This happens since pH influences the surface charge of both catalysts, affecting organic pollutant adsorption, as can be deduced from the curves in Fig. 13**a** and Fig. 13**b**. Moreover, the increase in pH also affected the production of ·OH radicals due to the auto-decomposition of H_2_O_2_ and suppresses the possibility of generating Fe(OH)^+^ species, which occurred at acidic pH and are more effective than Fe^2+^ (Manu and Mahamood 2011). Another important finding concerns the crucial role of hydrogen peroxide. Generally, although TiO_2_ is capable of generating hydroxyl radicals on the surface, the rate at which they are generated is very low compared to that resulting from the photolysis of H_2_O_2_, whose efficiency can approach unity (Egerton and Purnama 2014). For this reason, comparing the results at pH 7 with and without H_2_O_2_ (see Fig. 13**c**), a decrease in performance was seen in the second case. Surprisingly, in the latter case, i.e., under neutral conditions and in the absence of hydrogen peroxide, there was a reversal in performance, which could highlight two different degradation mechanisms over TiO_2__SCS350 and Fe/TiO_2__SCS samples. As previously mentioned, titania is a well-known photocatalyst that can oxidize various compounds thanks to the production of ·OH radicals when the surface is irradiated with a certain wavelength. In the case of TiO_2__SCS350, the acidic pH and the presence of hydrogen peroxide have the role of degrading the pollutant more quickly and improving performance. By increasing the pH and removing H_2_O_2_, a deterioration was seen but promising results were obtained, reaching just under 80% after 360 min. In contrast, the Fe/TiO_2__SCS sample exhibited a completely different behavior when the hydrogen peroxide was removed. This supports the fact that in this case, the catalyst is capable of performing a kind of photo-Fenton-like process. Indeed, in this type of process, both Fe^2+^ and H_2_O_2_ are reactants that react under UV/visible radiation to form active oxidant species leading to the destruction of the pollutant compounds (Wadhah 2017; Zanchettin et al. 2021). Thus, by removing a reagent, the degradation reaction almost suffers an inhibiting effect due to the lack of H_2_O_2_. Finally, it must be remembered that the presence of carbon in the structure or other carbon species could help prevent electron recombination in titania, as observed in another work (Kavitha and Devi 2014). Therefore, the additional species detected by the XPS analysis in the TiO_2__SCS350 sample might have had a positive effect.

## Conclusions

The removal of paracetamol from wastewater, before its discharge into the environment, is a crucial step to preserve the environmental quality and avoid harmful health effects. This work aimed to propose a novel and unconventional method (solution combustion synthesis) for the synthesis of mesostructured titania catalysts with enhanced performances toward the degradation of paracetamol. The temperature employed in the synthesis was found a crucial parameter in obtaining a highly defective and active TiO_2_ sample. Furthermore, an iron-containing sample was successfully obtained in a one-pot step, exhibiting the presence of metal iron species which played an important role in the paracetamol degradation, as revealed during the tests. The solution combustion synthesis (SCS) proved to be highly promising since it allowed to obtain high surface areas and defective structures, simply by mixing a precursor and a fuel, thus eliminating the use of numerous chemicals and equipment, providing a more sustainable, green, cost-effective, and non-time-consuming alternative with less impact on the environment. Concerning paracetamol removal, Fe/TiO_2__SCS demonstrated greater removal efficiency than the conventional P25, by reaching complete degradation in 1 h and following a pseudo-first-order kinetic model. Under near-neutral conditions, a different abatement mechanism was found in TiO_2__SCS350 and Fe/TiO_2__SCS, however, achieving a high percentage of removal in all cases. Overall, the presented results showed the potential of this uncommon technique to synthesize pure and iron-containing mesoporous titania, with a possible application even on a large scale.

### Supplementary Information

Below is the link to the electronic supplementary material.

Supplementary file1 (DOCX 1315 KB)

## Data Availability

All data generated or analyzed during this study will be provided on reasonable request.
