# Supplementary material for: Mesoporous TiO2 and Fe-containing TiO2 prepared by solution combustion synthesis as catalysts for the photodegradation of paracetamol
Source: Environ Sci Pollut Res Int. 2024 May 17;31(25):36861–81. doi: 10.1007/s11356-024-33575-5 (PMC11182819; doi:10.1007/s11356-024-33575-5)
Supplement: Supplementary file 1 — Supplementary file1 (DOCX 1315 KB) [file 11356_2024_33575_MOESM1_ESM.docx]

SUPPORTING INFORMATION

**Mesoporous TiO_2_ and Fe-containing TiO_2_ prepared by solution combustion synthesis as catalysts for the photodegradation of paracetamol**

Nadia Grifasi, Fabio Deorsola, Debora Fino, and Marco Piumetti^*^

Department of Applied Science and Technology, Corso Duca Degli Abruzzi, 24, 10129, Turin, Italy;

*****Correspondence: [marco.piumetti@polito.it](mailto:marco.piumetti@polito.it) (M. P.); Tel.: +39 0110904753

**Index**

**Fig. S1** Adsorption-desorption isotherms of P25 evaluated from N_2_-physisorption at -196 °C

**Fig. S2** X-Ray Diffraction pattern of P25 in the range of 2θ between 20-90 °

**Fig. S3** XPS spectra deconvolution of Ti 2p (**a**), O 1s (**b**) and C 1s (**c**) of the commercial TiO_2_ Degussa (P25)

**Fig. S4** Variation of H_2_O_2_ spectra as a function of its concentration

**Fig. S5** UV-vis spectra of the system with 10 ppm of PCT under acidic conditions (pH 3) in the presence of H_2_O_2_ under the exclusion of light source (dark conditions) for all the samples investigated

**Fig. S6** Diffractograms comparison between the fresh and the spent TiO_2__SCS350 catalyst

**Fig. S7** Comparison of the Ti 2p, O 1s, and C 1s spectra between the fresh and the spent TiO_2__SCS350 catalyst

**Fig. S8** Paracetamol degradation spectra on P25 under UV light source (302 nm), initial concentration of paracetamol = 10 ppm, catalyst dosage = 2 g L^-1^, pH 3, H_2_O_2_ 2.78 mM, temperature of 25 °C

**Fig. S9** Diffractograms comparison between the fresh and the spent Fe/TiO_2__SCS throughout the photocatalytic test

**Fig. S10** XPS Fe 2p spectra of the spent Fe/TiO_2__SCS catalyst collected at 5 minutes (**a**), 30 minutes (**b**), and 60 minutes (**c**)

**Table S1** Textural properties of the commercial TiO_2_ Degussa (P25)

**Table S2** Atomic concentration of the species detected from the spectra deconvolutions of the commercial TiO_2_ Degussa (P25)

**Table S1** Textural properties of the commercial TiO_2_ Degussa (P25)

| **Sample** | **SSA^a^ [m^2^ g^-1^]** | **Vp^b^ [cm^3^ g^-1^]** | **Dp^c^ [nm]** | **Crystallite size^d^ [nm]** |
| --- | --- | --- | --- | --- |
| P25 | 52 | 0.19 | 12 | 31 |

^a^ Specific surface area (SSA) evaluated according to the Brunauer–Emmett–Teller (BET) method.

^b^ Total Pore Volume (VTP) evaluated according to the Barrett–Joyner–Halenda (BJH) method during the desorption phase.

^c^ Average pore diameter Dp evaluated according to the Barrett–Joyner–Halenda (BJH) method during the desorption phase.

^d^ Average crystallite dimension evaluated according to the Scherrer equation.





**Fig. S1** Adsorption-desorption isotherms of P25 evaluated from N_2_-physisorption at -196 °C





**Fig. S2** X-Ray Diffraction pattern of P25 in the range of 2θ between 20-90 °



**Fig. S3** XPS spectra deconvolution of Ti 2p (**a**), O 1s (**b**) and C 1s (**c**) of the commercial TiO_2_ Degussa (P25)

**Table S2** Atomic concentration of the species detected from the spectra deconvolutions of the commercial TiO_2_ Degussa (P25)

| Element | Species detected | P25 |
| --- | --- | --- |
| O *1s* | Lattice | 71.9% |
|  | OH | 28.1% |
|  | C=O | - |
| Ti *2p* | Ti^4+^ | 96.7% |
|  | Ti^3+^ | 3.3% |
|  | Ti^2+^ | - |
| C *1s* | Ti-C-O | - |
|  | C-O-C | 28.2% |
|  | Ti-O-C=O,  C=O | 4.9% |
|  | C-C,  C-H | 66.9% |


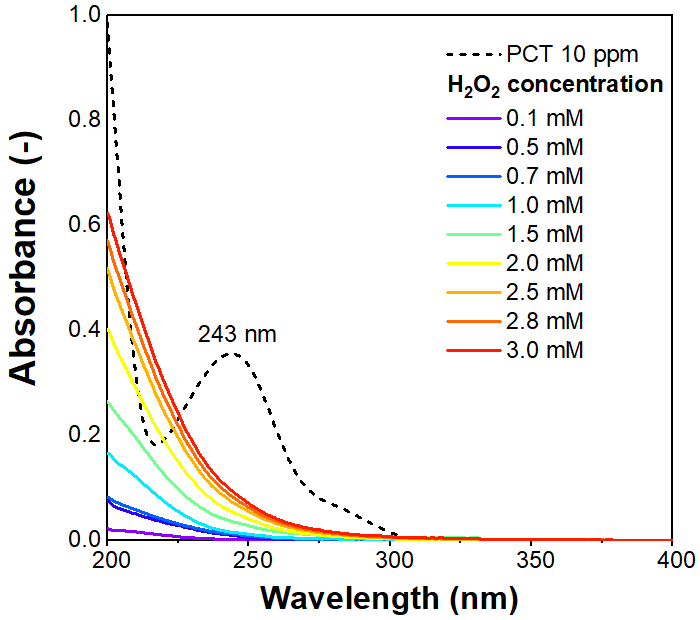


**Fig. S4** Variation of H_2_O_2_ spectra as a function of its concentration



**Fig. S5** UV-vis spectra of the system with 10 ppm of PCT under acidic conditions (pH 3) in the presence of H_2_O_2_ under the exclusion of light source (dark conditions) for all the samples investigated





**Fig. S6** Diffractograms comparison between the fresh and the spent TiO_2__SCS350 catalyst



**Fig. S7** Comparison of the Ti 2p, O 1s, and C 1s spectra between the fresh and the spent TiO_2__SCS350 catalyst





**Fig. S8** Paracetamol degradation spectra on P25 under UV light source (302 nm), initial concentration of paracetamol = 10 ppm, catalyst dosage = 2 g L^-1^, pH 3, H_2_O_2_ 2.78 mM, temperature of 25 °C





**Fig. S9** Diffractograms comparison between the fresh and the spent Fe/TiO_2__SCS throughout the photocatalytic test





**Fig. S10** XPS Fe 2p spectra of the spent Fe/TiO_2__SCS catalyst collected at 5 minutes (**a**), 30 minutes (**b**), and 60 minutes (**c**)
